# The Impact of Social Media on Dissemination and Implementation of Clinical Practice Guidelines: A Longitudinal Observational Study

**DOI:** 10.2196/jmir.4414

**Published:** 2015-08-13

**Authors:** Pushpa Narayanaswami, Gary Gronseth, Richard Dubinsky, Rebecca Penfold-Murray, Julie Cox, Christopher Bever Jr, Yolanda Martins, Carol Rheaume, Denise Shouse, Thomas SD Getchius

**Affiliations:** ^1^ Beth Israel Deaconess Medical Center Department of Neurology Harvard Medical school Boston, MA United States; ^2^ University of Kansas School of Medicine Department of Neurology Kansas City, KS United States; ^3^ American Academy of Neurology Minneapolis, MN United States; ^4^ VA Maryland Health Care System's MS Center of Excellence Department of Neurology University of Maryland School of Medicine Baltimore United States; ^5^ Dana Farber Cancer Institute Survey and Data Management Core Harvard Medical School Boston, MA United States

**Keywords:** information dissemination, social media, multiple sclerosis, complementary medicine, medicine, complementary, therapy, complementary, alternative medicine, alternative therapies, clinical practice guidelines, dissemination and implementation

## Abstract

**Background:**

Evidence-based clinical practice guidelines (CPGs) are statements that provide recommendations to optimize patient care for a specific clinical problem or question. Merely reading a guideline rarely leads to implementation of recommendations. The American Academy of Neurology (AAN) has a formal process of guideline development and dissemination. The last few years have seen a burgeoning of social media such as Facebook, Twitter, and LinkedIn, and newer methods of dissemination such as podcasts and webinars. The role of these media in guideline dissemination has not been studied. Systematic evaluation of dissemination methods and comparison of the effectiveness of newer methods with traditional methods is not available. It is also not known whether specific dissemination methods may be more effectively targeted to specific audiences.

**Objective:**

Our aim was to (1) develop an innovative dissemination strategy by adding social media-based dissemination methods to traditional methods for the AAN clinical practice guidelines “Complementary and alternative medicine in multiple sclerosis” (“CAM in MS”) and (2) evaluate whether the addition of social media outreach improves awareness of the CPG and knowledge of CPG recommendations, and affects implementation of those recommendations.

**Methods:**

Outcomes were measured by four surveys in each of the two target populations: patients and physicians/clinicians (“physicians”). The primary outcome was the difference in participants’ intent to discuss use of complementary and alternative medicine (CAM) with their physicians or patients, respectively, after novel dissemination, as compared with that after traditional dissemination. Secondary outcomes were changes in awareness of the CPG, knowledge of CPG content, and behavior regarding CAM use in multiple sclerosis (MS).

**Results:**

Response rates were 25.08% (622/2480) for physicians and 43.5% (348/800) for patients. Awareness of the CPG increased after traditional dissemination (absolute difference, 95% confidence interval: physicians 36%, 95% CI 25-46, and patients 10%, 95% CI 1-11) but did not increase further after novel dissemination (physicians 0%, 95% CI -11 to 11, and patients -4%, 95% CI -6 to 14). Intent to discuss CAM also increased after traditional dissemination but did not change after novel dissemination (traditional: physicians 12%, 95% CI 2-22, and patients 19%, 95% CI 3-33; novel: physicians 11%, 95% CI -1 to -21, and patients -8%, 95% CI -22 to 8). Knowledge of CPG recommendations and behavior regarding CAM use in MS did not change after either traditional dissemination or novel dissemination.

**Conclusions:**

Social media-based dissemination methods did not confer additional benefit over print-, email-, and Internet-based methods in increasing CPG awareness and changing intent in physicians or patients. Research on audience selection, message formatting, and message delivery is required to utilize Web 2.0 technologies optimally for dissemination.

## Introduction

Evidence-based clinical practice guidelines (CPG) are statements that assist clinical decision making by providing recommendations for optimizing patient care for a specific clinical question. They are informed by a systematic review of evidence and an assessment of the benefits and harms of the evaluated care options [1,2]. Dissemination of CPG recommendations is commonly undertaken by publishing the CPG in a peer-reviewed journal, sending email or paper notices to physicians, and advertising through news media outlets [3].

There is a large amount of medical information available on the Internet and social media. There is some evidence that social media are useful for disseminating medical information, but the quality and accuracy of information vary and may even be misleading [4-22]. Specific to neurology, one report noted accurate demonstration of the Epley maneuver for benign paroxysmal positional vertigo on YouTube [23]. Web-based tools and social media networks (eg, Facebook, YouTube, LinkedIn, Twitter, Google+) have the potential to reach large audiences in a short time span for rapid communication of CPG recommendations [5]. In a recent survey by the Pew Research Center, over two-thirds of Americans reported using the Internet for health and fitness information [4]. This figure did not vary substantially across demographic subgroups. However, there is scant research on the effectiveness of the use of social media platforms in dissemination and implementation of CPG recommendations. The use of targeted approaches within social media platforms to direct and focus guideline dissemination efforts to specific populations has not been evaluated [24].

The American Academy of Neurology (AAN) has developed CPGs since 1989 and has employed a formal dissemination program since 1999 to raise awareness and enable implementation of CPG recommendations. In this study, we evaluate the effectiveness of social media for disseminating recommendations of the recently developed CPG, “Complementary and alternative medicine in multiple sclerosis” (“CAM in MS”) [25]. Complementary and alternative medicine (CAM) use is widely prevalent in 33-80% of patients with multiple sclerosis (MS). These patients often do not discuss this use with their physicians [26-33]. A study of information sources used by people with MS revealed that the Internet was the first source of general health information in 73% and for MS-specific information in 59% [34]. Because this is the first CPG on CAM use in MS, we used it to study dissemination tactics, with special emphasis on social media use, to inform future dissemination efforts for CPGs.

The specific aims of this study were to (1) develop an innovative dissemination strategy by adding novel, social media−based methods to traditional dissemination methods for the CPG “CAM in MS”, (2) evaluate whether the addition of social media−based methods improves CPG awareness and knowledge of CPG recommendations in the two target audiences of patients and physicians/clinicians (referred to herein as “physicians”), and (3) evaluate whether the addition of social media−based methods improves the implementation of CPG recommendations. Implementation in this context is defined as the adoption and integration of evidence-based health interventions to change practice [35].

## Methods

### Study Definitions and Design

This was a longitudinal observational study, using quantitative survey methods (AHRQ IR18HS022004-01; Grants.gov tracking 11129815). The study was determined to be exempt from the need for ethical review and approval by the Committee on Clinical Investigations, Beth Israel Deaconess Medical Center, Boston. We have substantially complied with the Workgroup for Intervention Development and Evaluation Research (WIDER) recommendations for reporting research evaluating behavioral interventions [36]. The intervention and co-intervention were traditional and novel dissemination methods, respectively, to disseminate the AAN CPG “CAM in MS”. We defined traditional methods as dissemination using print-, email-, and Internet-based methods. This included publication of the CPG in *Neurology*, the official journal of the AAN; issuance of a news release (electronic release and public relations pitch to approximately 700 science and medical reporters); development of clinician and patient summaries, a clinical case example, and presentation slide set of CPG content; news articles in AAN*news, Neurology Today*, and *Neurology Now* (official publications of the AAN); and electronic notices to 26,965 AAN members, consisting of all-member emails, an AAN *e-News* announcement, and an announcement in the AAN quarterly Leadership Update and highlights of the CPG on AAN.com.

We defined novel methods as dissemination through social media platforms. These included an audio podcast on *Neurolog*y; videos for patients and physicians posted on YouTube, with links to the CPG on AAN.com; and Facebook, Twitter, LinkedIn, and YouTube digital advertising (where feasible, targeted audiences endorsing an interest in CAM use in MS and related terms were selected; Multimedia Appendix 1). For digital advertisements, the research team treated two of the more popular channels as primary (Facebook and Twitter) and two as secondary (LinkedIn and YouTube) for the purpose of allocating funding over the 90-day dissemination period. Over three periods of 30 days each, the team tested digital advertisements across all four platforms, mixing and matching written text (copy) and visual images, and evaluated the number of impressions (the number of times the advertisement was “served”, or seen) for each advertisement. The goal was first to identify which copy would produce the most impressions in the first period and which image would product the most impressions in the second period, and then to promote the most impactful copy and image for the final period. We also held a chat on Twitter in partnership with *TIME* Magazine, the National Multiple Sclerosis Society (NMSS), and Beth Israel Deaconess Medical Center in Boston between 12 noon and 1 p.m. Eastern Time on August 28, 2014. A moderator led the Twitter chat by asking questions regarding general information about MS, what treatments are available, what the evidence indicates, and where future research should be directed. Informational emails were sent during both traditional and novel dissemination periods to patient organizations (Multiple Sclerosis Association of America, Multiple Sclerosis Foundation, NMSS). These emails informed the organizations of the availability of the guideline and patient summaries, provided links to the guideline, gave a summary of the key guideline recommendations, and requested their support in disseminating the guideline to their members.

**Figure 1:**
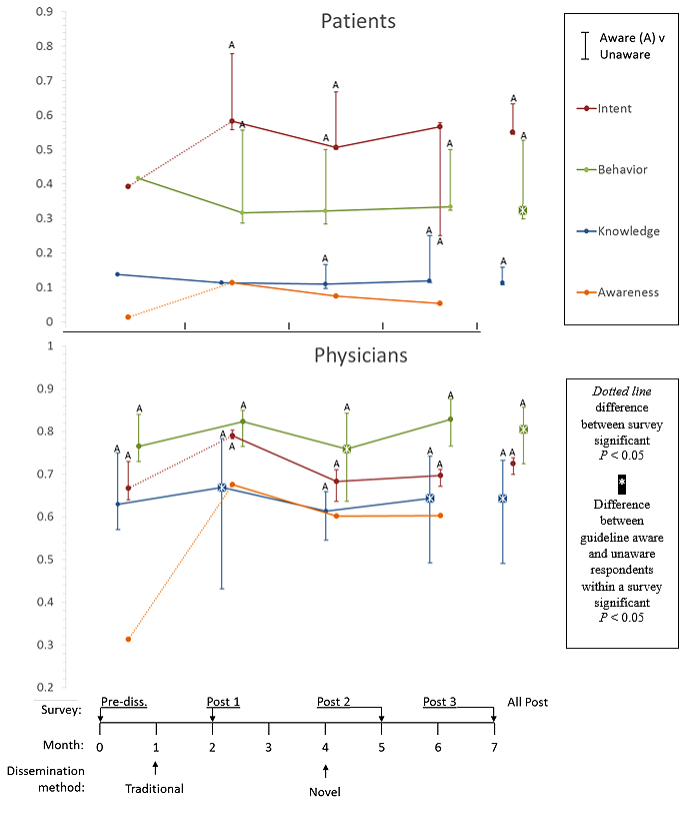
Timeline of dissemination (horizontal axis), CPG awareness, correct CAM knowledge, intent to discuss CAM, and behavior for patients and physicians. Patient results are represented in the upper set of graphs, and physician results in the lower set. The horizontal axis represents time in months and delineates the point of the traditional and novel dissemination efforts and each of the 4 study surveys: pre-dissemination, post-traditional dissemination (post-1), post-novel dissemination (post-2), the results 6 months after traditional dissemination (post-3), and combined results of all post-dissemination surveys. The vertical axis represents the percentage of respondents. The outcome variables (awareness, knowledge, intent, and behavior) are represented by orange, blue, green, and maroon lines, respectively. Dotted lines indicate significant differences between the 2 surveys. The vertical bars represent within-surveys comparison of unaware and aware respondents for each outcome variable. Starred boxes indicate significant differences.

The study design and timeline are summarized in Figure 1. We assessed outcomes using survey questionnaires developed for this study. We conducted four surveys in each of the two target populations (physicians, patients). First, we conducted a pre-dissemination survey 1 month before dissemination of the CPG (Feb 2014). The CPG was then published in *Neurology* in March 2014 and simultaneously disseminated using traditional methods. Three months after traditional dissemination (June 2014), the guideline was disseminated using novel methods. We conducted the second and third surveys, post-dissemination survey-1 and post-dissemination survey-2, 1 month after traditional (April 2014) and novel (July 2014) dissemination, respectively. Finally, 6 months after traditional dissemination (Sept 2014), we conducted post-dissemination survey-3.

### Study Population

The study participants were drawn from two populations: physicians who treated patients with MS, and patients with MS / caregivers of patients with MS. We identified physician participants from the AAN membership database using the following criteria: neurologist or neurology advanced practice provider with a focus in MS practice, residing in the United States, aged ≤68 years, with mailing and email addresses, who had not received an AAN survey in the prior 6 months. A random sample of 620 physicians was drawn from this population for the pre-dissemination survey. For each subsequent survey, physicians surveyed in the previous survey(s) were excluded, and 620 physicians were randomly selected from the remaining population.

Patient participants were identified from the database of subscribers to *Neurology Now*, an official bimonthly publication of the AAN for patients. Of 23,568 subscribers who self-identified as interested in MS, 10,818 subscribers with email addresses were used as the final dataset. From this dataset, 200 patients were randomly selected for the pre-dissemination survey. For each subsequent survey, patients surveyed in the previous survey(s) were excluded, and 200 patients were randomly selected from the remaining population.

### Surveys and Data Collection

A team of investigators and survey methodologists developed the surveys in accordance with the American Association of Public Opinion Research evidence-based, best practices for survey research [37] (see Multimedia Appendix 2 for survey development methods and survey data collection procedure). For patients, the surveys evaluated their knowledge and use of CAM in general, the specific CAM therapies discussed in the CPG, reasons for CAM use, and perceived efficacy of CAM. We explored potential confounders: frequency of Internet use and the presence of any disability interfering with Internet use. In the physician surveys, we assessed knowledge, attitudes, and behavior regarding CAM in MS.

Data collection was performed by the Dana-Farber/Harvard Cancer Center Survey and Data Management Core, who used DatStat Illume. Survey responses were assigned a numeric code and were stored in a separate database without identifying information.

### Statistical Analysis

#### Outcome Measures

The outcome measures were a change in knowledge, intent, and behavior, all with respect to CAM in MS. Knowledge of CAM was assessed by six survey questions regarding the effectiveness of magnetic therapy, oral cannabis extract, smoked cannabis, ginkgo biloba, hyperbaric oxygen, and bee venom therapy, all of which were discussed in the CPG. A 5-point ordinal scale was used for responses (Multimedia Appendix 3). For analysis of change in knowledge, we used the response to the question: “Taking ginkgo biloba orally is effective for improving memory in people with MS”. Change in intent was defined as patients’ intent to discuss CAM use with their physicians and physicians’ intent to discuss CAM use with their patients. Change in behavior was defined as patients’ discussing CAM with their physicians and physicians’ recommending that their patients start or stop any CAM therapy. General attitudes (beliefs and views towards CAM in MS) were also explored.

The ordinal responses were collapsed into two categories: (1) agree and somewhat agree, and (2) somewhat disagree, disagree, and not sure. We believed that this question measured knowledge most accurately because responses to it were unlikely to be biased by prior opinions as compared with responses likely to be elicited by the therapies assessed in the other questions.

The primary outcome was the difference in respondents’ intent to discuss CAM for the two study populations (ie, physicians and patients) with their respective patients/physicians, after novel dissemination (post-dissemination survey-2) as compared with that after traditional dissemination (post-dissemination survey-1). Secondary outcomes were (1) awareness of the CPG, (2) knowledge of CPG recommendations, (3) behavior regarding CAM use in MS (these three outcomes were measured at baseline, ie, pre-publication/dissemination, and after the two dissemination periods, traditional and novel, ie, between-surveys differences), and (4) knowledge, intent, and behavior, measured in CPG-aware physicians and patients. All four outcomes were compared with those unaware of the CPG, within each of the four surveys (ie, within-survey differences). Because behavior changes take time and may not be captured in the time frame of this study and because knowledge may not translate into action, we selected intent to change a priori as a surrogate outcome of behavior. The underlying framework for this choice is the Theory of Planned Behavior that is used to predict behavioral intention and behavior. Intention leads to behavior when attitudes are strong and perceived behavioral control is high [38].

#### Sample Size Estimations

##### Physicians

Assuming a 10% responder rate per survey and a 20% change in intent as clinically important, we surveyed 620 physicians in each of four surveys to obtain 62 responses, for an 80% chance (β) of detecting this difference at a significance level alpha=.05.

##### Patients

Assuming a 20% responder rate per survey and a 10% change in intent as clinically important, we surveyed 200 patients in each of four surveys to obtain 19 responses, for an 80% chance (β) of detecting this difference at a significance level alpha=.05.

### Data Analysis

As the measure of effect, we used the absolute difference (AD) between surveys in the proportion of respondents who (1) were aware of the CPG, (2) had correct knowledge of the recommendations discussed in the CPG, (3) endorsed intent to discuss CAM use in MS, and (4) endorsed a recent behavior change regarding CAM use. Secondary analysis stratified respondents within each survey into CPG-aware and CPG-unaware groups. We compared intent, knowledge, and behavior of respondents in CPG-aware and CPG-unaware groups (within survey differences). Precision was measured with 95% confidence intervals (CI). We did not adjust for multiple comparisons for the secondary outcomes.

## Results

### Dissemination Efforts

The results of the traditional and novel dissemination efforts are summarized in Multimedia Appendix 4.

### Results of Surveys

We surveyed a total of 2480 physicians (620 per survey) and 800 patients (200 per survey) across four surveys. The *total response rate* across all four surveys for physicians was 25.08% (622/2480), and for patients was 43.5% (348/800), over twice our predicted rate, and was stable across all four surveys (physicians 23-27%, patients 42-45%) (Table 1). Approximately equal numbers of participants responded to mailed (paper) surveys and email surveys (mailed survey responses: physicians 52-60%, mean across surveys 55%; patients 47-54%, mean across surveys 50.5%). Response rate denominators to individual questions may vary slightly because of incomplete responses. There was no substantial difference between respondent and nonrespondent physicians across the surveys in age or sex (Table 1). Comparison of patient respondents to nonrespondents was not possible, as demographic data were not available for patients from the *Neurology Now* database.

**Table 1:** Characteristics of surveyed patients and physician (95% confidence interval rounded to nearest whole percent).

		Physicians, % (95% CI)	Patients, % (95% CI)
Sampled, n		2480	800
Responded, n		622	348
**Age**			
	Age sample years, mean (SD)	52.0 (9.4)	
	Age respondents years, mean (SD)	52.1 (9.8)^a^	54.8 (11.7)
**Women**			
	Sampled	31.4 (30-33)	
	Respondents	34.4 (31-38)^b^	80.2 (76-84)
White		74.4 (71-78)	86.8 (83-90)
Internet use <daily		4.7 (3.3-6.6)	25.6 (21-30)
Practice >15 years		61.9 (58-66)	
Care for >50 patients with MS		57.4 (54-61)	
University-based practice		21.1 (18-24)	
Ever recommend any CAM		79.6 (76-83)	
MS duration >10 years			56.3 (51- 61)
College diploma			55.4 (50- 61)
Walking without assistance			56.1 (51-61)
No difficulty with hand use			43.9 (38-50)
Normal vision			49.2 (44-55)
Ever used any CAM			70.9 (66-76)

^a^Difference in mean age between sample and respondents with 95% CI -0.1 years, -0.93 to 0.73.

^b^Difference in percent women between sample and respondents with 95% CI 3%, -1.1 to 7.2.

### Demographics and General Attitudes to Complementary and Alternative Medicine in MS

#### Physician Respondents

The mean age (standard deviation) was 52.1 (SD 9.8) years (range across surveys 50.7; SD 10.3 to 52.2 years; SD 9.7). Over a third (199/578, 34.4%; range across surveys 31-38%) were women, and three-quarters (445/598, 74.4%; range 69-82%) were white. The practice focus was 31.7% (185/583) group practice, 21.4% (123/583) university-based practice, 18.7% (109/583) solo practice, and 17.0% (99/583) multispecialty practice, and the remaining were in health maintenance organization (HMO), government, or other practice types. A total of 62.1% (362/583) of physicians had been in practice for >15 years, and 12.5% (73/585) for <5 years. Over half the physicians (337/587, 57.4%) were treating more than 50 patients with MS, and almost all (572/600, 95.3%) used the Internet daily (Table 1).

Approximately half the physicians reported routinely discussing CAM with their patients (307/598, 51.3%, range across surveys 48-56%). Very few (23/607, 3.8%; range 1-6%) reported being unaware of CAM use in their patients, and 79.6% (483/607) of physicians said they recommended some form of CAM to their patients (range 76-83%). Yoga (total 385/607, 63.4%, mean across surveys 61%), massage therapy (total 417/607, 68.7%, mean 52%), and acupuncture (total 408/607, 67.2%, mean 51%) were the CAM therapies that physicians considered most useful. Physicians were also most likely to recommend these three CAM therapies (mean across surveys massage 49%, yoga 48%, acupuncture 47%). Half (313/607, 51.6%) of the physicians reported that their patients used marijuana, but only 6.9% (42/607) stated that they would recommend it.

#### Patient Respondents

The mean age was 54.8 (SD 11.7): range across surveys 54.2 (SD 12.2) to 55.6 (SD 11.6) years. A total of 80.2% (243/303; range 75-87% across surveys) were women. Three-quarters (236/317, 74.4%, range 68-83%) used the Internet daily, and only 9.5% (30/317; range 8-11%) used the Internet once a week or less. Some disability in use of the Internet was reported by just under a quarter (68/312, 21.8%; range 18-28%). Over half the patients reported MS duration of >10 years (171/304, 56.3%; range across surveys 53-60%). MS duration was <5 years in 16.8% (51/304; range 14-19%), 55% (range 48-62%) had at least a college diploma (college diploma: 82/221, 37.1%; some post-graduate education; 28/275, 10.2%, post-graduate diploma: 58/245, 23.7%), over half (169/301, 56.1%; range 49-67%) were ambulatory without assistance, half (148/301, 49.2%; range 37-56%) had normal vision, and under half (133/303, 43.9%; range 34-47%) had no difficulty with use of their hands.

Across surveys, 67.7% (212/313) of patient respondents had heard of CAM (55/79, 70% in the pre-dissemination survey; 49/77, 64%, 46/80, 58%, and 62/77, 81% in the three post-dissemination surveys, respectively) (Table 1). The most common therapies patients reported discussing with their physicians were acupuncture (26/233. 11.2%), dental amalgam removal (21/233, 9.0%), and bee sting therapy (16/233, 6.9%).

### Comparison of Results of Pre-dissemination, Post-Traditional Dissemination, and Post-Novel Dissemination Surveys

#### Awareness of the clinical practice guideline “Complementary and Alternative Medicine in Multiple Sclerosis”

In the pre-dissemination survey, 31.3% (45/144) of physicians were aware of a guideline on CAM in MS. In physicians, there was a significant increase in awareness of the CPG after traditional dissemination (pre-dissemination survey vs post-dissemination-1 survey, awareness AD 36%, 95% CI 25-46%). Although the heightened awareness of the CPG persisted after novel dissemination, there was no further increase in awareness after novel dissemination (AD post-dissemination -1 vs post-dissemination-2 surveys -7%, 95% CI -18 to 4) (Figure 1).

Only 0.1% (1/76) patients was aware of the upcoming CPG in the pre-dissemination survey. There was a statistically significant increase in awareness of the CPG after traditional dissemination that did not change after novel dissemination, although the absolute number of aware patients was small (AD pre-dissemination vs post-dissemination-1: 10%, 95% CI 1-11; post-dissemination-1 vs post-dissemination-2: -4%, 95% CI -6 to 14).

#### Intent to Discuss Complementary and Alternative Medicine

The intent to discuss CAM (for physicians, with their patients with MS, and for patients/caregivers, with their physicians) increased significantly in both groups after traditional dissemination (AD pre-dissemination vs post-dissemination-1: physicians were 12%, 95% CI 2-22, patients were 19%, 95% CI 3-33). Our primary outcome measure did not change: the proportion of either patients or physicians reporting an intent to discuss CAM did not increase after novel dissemination as compared with that after traditional dissemination (AD post-dissemination-1 vs post-dissemination-2: physicians were -11%, 95% CI -1 to -21, patients were -8%, 95% CI -22 to 8).

Stratified analysis of the differences in intent between CPG-aware and CPG-unaware respondents within each survey revealed that awareness of the CPG was not associated with an increase in physicians’ reporting of an intent to discuss CAM with their patients (intent change, physicians, AD aware vs unaware, all surveys: 4%, 95% CI -5 to 13). CPG-aware patients in post-dissemination surveys were also not significantly more likely to report an intent to discuss CAM with their physicians (intent change, patients, AD aware vs unaware, all surveys: 9%, 95% CI -14 to 28). However, due to the small number of aware patients, the precision of the estimated difference is low.

#### Knowledge of Clinical Practice Guideline Recommendations

Correct knowledge of the recommendations discussed in the CPG did not significantly change in either physicians or patients after traditional or novel dissemination as compared with pre-dissemination (physicians AD pre-dissemination vs post-dissemination-1: 4%, 95% CI -7 to 15; post-dissemination-1 vs post-dissemination-2: -6%, 95% CI -16 to 5; patients AD pre-dissemination vs post-dissemination-1: -2%, 95% CI -13 to 8; post-dissemination-1 vs post-dissemination-2: -4%, 95% CI -14 to 6).

In the stratified analysis, physicians who were aware of the CPG had better knowledge of CAM than physicians who were not aware of the CPG (knowledge change, physicians, AD aware vs unaware, all surveys: 24%, 95% CI 15-33). Patients who were aware of the CPG did not have significantly better knowledge than those who were not aware (knowledge change, patients, AD aware vs unaware, all post-dissemination surveys: 5%, 95% CI -6 to 27). However, because of the small number of aware patients, the precision of this estimate is low.

#### Behavior With Regard to Complementary and Alternative Medicine Therapies

The number of physicians who had recommended that their patients stop or start using any CAM therapy did not change significantly after either traditional or novel dissemination as compared with pre-dissemination; that is, there was no change in behavior (behavior change AD pre-dissemination vs post-dissemination-1: 5%, 95% CI -4 to 14; post-dissemination-1 vs post-dissemination-2: -6.4%, 95% CI -16 to 3). Across all surveys, physicians who were aware of the CPG made recommendations more frequently regarding the use of CAM therapies as compared with CPG-unaware physicians (AD physicians making recommendations, aware vs unaware, all surveys: 13%, 95% CI 6-21).

There was no significant increase in the proportion of patients who reported having recently discussed CAM with their physicians following traditional or novel dissemination (behavior change, patients, pre-dissemination vs post-dissemination-1, AD: 10%, 95% CI -6 to 26; post-dissemination-1 vs post-dissemination-2: 1%, 95% CI -14 to 15). However, across all surveys, CPG-aware patients were more likely to have discussed CAM with their physicians (AD patients, aware vs unaware, all surveys: 23%, 95% CI 1-44).

## Discussion

### Diffusion and Dissemination

The term *diffusion* is used by some authors to describe the distribution and unaided adoption of information, whereas *dissemination* refers to a more active process of communication to improve knowledge [39]. Other authors use the terms synonymously. Dissemination is defined as “the purposive distribution of information and intervention materials to a specific public health or clinical practice audience.” Dissemination research studies how “information about health promotion and care interventions is created, packaged, transmitted, and interpreted among important stakeholder groups” [40]. It is recognized that there is a gap between CPG development and the delivery of care in practice. Merely reading a CPG rarely leads to implementation of recommendations [39]. Active, effective dissemination of CPG recommendations to end users is essential for optimizing care delivery.

### Principal Results

In this study, about a third of the physicians were aware of the “CAM in MS” CPG before publication and dissemination, and almost three-quarters reported recommending CAM therapies in the pre-dissemination survey. Our traditional dissemination methods (print, email, and Internet) were successful in increasing awareness of the CPG in physicians and patients. Overall awareness of the CPG was low in patients despite a statistically significant increase between pre-dissemination and post-traditional dissemination. Traditional dissemination methods were also effective in increasing intent to discuss CAM in both physicians and patients. Despite increased awareness of the CPG, knowledge did not change in physicians across either dissemination method, although an increase in knowledge was noted in CPG-aware physicians as compared with those unaware of the CPG. Knowledge also did not change in patients with either dissemination method, regardless of whether they were aware of the CPG. A significantly greater proportion of CPG-aware physicians and CPG-aware patients reported discussing CAM use with their patients and physicians, respectively, across all the post-dissemination surveys (behavior change).

The lack of change in knowledge among physicians could be due to a ceiling effect in an already enriched population of physicians with interests in CAM and MS and a high baseline level of knowledge of the topic. For patients, the lack of increase in knowledge may have resulted from the overall low awareness. However, knowledge was no different in patients who were aware of the CPG and those who were unaware. It is difficult to draw any conclusions given the small number of aware patients. It is intriguing that intent changed in the absence of increased knowledge. Perhaps the questions used to capture knowledge of the CPG were not directly relevant to intent, which was broadly defined as intent to discuss CAM or intent to start/stop any CAM.

To our surprise, despite an apparently successful dissemination effort using novel media as measured by the reach of Facebook, Twitter, YouTube, and LinkedIn, there was no additional increase in awareness, intent (our primary outcome), knowledge, or behavior in either physicians or patients after social media dissemination efforts as compared with traditional methods. We used targeted advertising to try to reach audiences interested in MS and CAM, and we cast a fairly wide net. The reasons for apparent lack of effectiveness of social media as compared with traditional dissemination methods merit further study but may be related to several factors. First, the CPG may have been considered “old news” when disseminated through social media because traditional dissemination had already saturated the target audience. However, this does not fully explain the large numbers of “hits” that the CPG received on multiple social media networks. Second, despite the fact that the CPG received wide attention through social media advertising, the audience did not follow through by clicking the links to the CPG, and hence, did not remember the CPG or its contents. Third, the respondents of the surveys may not have been users of social media. Finally, social media may not be a useful tool for disseminating CPG recommendations. In a previous study, a social media marketing campaign on public awareness of hypertension did not change knowledge among participants, and the authors suggested that the target of dissemination efforts should be medical professionals in order to increase patient awareness at the point of care [41].

### Limitations

Because of the lack of availability of demographics between patient respondents and nonrespondents, we could not evaluate potential confounders. We chose a “between-participants” design rather than a “within-participants” design to avoid the learning and bias that would be expected in serial surveys of the same respondents. Although a comparison of respondents across surveys did not reveal significant differences (data not shown), a difference cannot be excluded with certainty, with resultant effects on our outcome measures.

Finally, contamination between traditional and novel methods cannot be excluded, as we did not have any control over how the CPG, when published, would be shared by the target audience. It is possible that contamination may explain some of the lack of effect of social media. We also recognize that our physician population was an inherently enriched one. An alternative study methodology may be to use a more controlled setting than the real-world setting that we studied. This could potentially be done with a pre- identified study population randomized into two cohorts of traditional dissemination and social media dissemination, with instructions to access the information only through the dissemination method to which they were randomized. However, this would also have limitations, including those of an artifactual setting that may not reflect real-world results. As part of traditional dissemination, a news report of the CPG was published in *Neurology Now*. Because the patient respondents were chosen from the subscriber database of this publication, it is possible that we may have enriched our patient population as well. However, we saw only slight increases in awareness after traditional dissemination, suggesting that patient participants may not have been enriched. A problem that the AAN anticipated was the ability to control the conversation and the perception of the news media interpretation of the recommendation for use of cannabis extracts. AAN’s communications team works with trusted media reporters to promote accurate messaging of the CPG recommendations, to minimize misrepresentation of the evidence, but recognizes that this is not foolproof.

### Conclusions

Our results are important in planning future dissemination efforts. Although we did not detect a difference in the effectiveness of the social media−based interventions as compared with the traditional methods, social media were useful in reaching large numbers of the public. We are planning subgroup analyses that may inform future targeted dissemination efforts to those populations that use social media most effectively. The diffusion of innovations model by Rogers is often utilized in disseminations research [42], and it has been suggested that social media platforms such as YouTube, integrated into these models, may be useful [43]. The term “Web 2.0” has been applied to the interactive Internet experience of today [44]. Access, relevance, and credibility have been described as the three critical criteria in using Web 2.0 technologies for dissemination [45]. Partnerships with commercial technology companies, utilization of rapid and adaptive designs to identify successful strategies for user engagement, and iterative evaluation of their efficacy have been recommended to effectively harness Web 2.0 for dissemination [9]. Further research is needed on methods to effectively harness social media platforms that have the potential to easily and inexpensively reach large audiences. This includes audience selection, message formatting and delivery, and other messaging characteristics. Focus group discussions and surveys/interviews of the target audiences (physicians and patients) may provide valuable input to refine social media use in dissemination of CPGs. Research is also needed on milestones and metrics to measure implementation of CPG recommendations.

## Appendix

Multimedia Appendix 1 Audience selection for social media dissemination.

Multimedia Appendix 2 Survey development methods and survey data collection procedure.

Multimedia Appendix 3 Surveys used in the study.

Multimedia Appendix 4 Results of dissemination efforts.

## Abbreviations

AAN: American Academy of Neurology
AD: absolute difference
CAM: complementary and alternative medicine
“CAM in MS”: “Complementary and alternative medicine in multiple sclerosis”
CPG: clinical practice guideline
HMO: health maintenance organization
MS: multiple sclerosis
NMSS: National Multiple Sclerosis Society
