# Supplementary material for: The Impact of Social Media on Dissemination and Implementation of Clinical Practice Guidelines: A Longitudinal Observational Study
Source: J Med Internet Res. 2015 Aug 13;17(8):e193. doi: 10.2196/jmir.4414 (PMC4736287; doi:10.2196/jmir.4414)
Supplement: Multimedia Appendix 1 [file jmir_v17i8e193_app1.pdf]

Multimedia Appendix 1: Novel Dissemination Methodology

Process for Targeting Audiences through Social Media Advertising

Determined the demographics and interests to use for Facebook and applied those across social media platforms (however, more terms available for use on Facebook than on other platforms).

- Chose ages 18 and older for demographics
- Chose numerous applicable/related terms for interests
- Categorized interests/terms as follows:
  - Medical/patient advocacy organizations
  - Health science
  - General disease-related topics
  - Signs/symptoms
  - General complementary and alternative medicine (CAM)–related topics
  - Therapies
- Subcategorized terms/interests for therapies in two ways: by topic and by level of evidence
  - By topic:
    - General
    - Dietary supplements
    - Energy medicine
    - Herbs
    - Manipulative and body-based practices
    - Mind–body medicine
  - By level of evidence:
    - Therapies with recommendations
    - Therapies with Level U finding
    - Therapies mentioned in the guideline as not having been studied in the guideline
- Decided to modulate the ad audience so as to spend allocated funds judiciously (not go “too wide” or “too narrow”). To do this, we assigned three tiers of significance to the set of terms
  - Tier 1: terms with greatest relevance to the population of interest
    - Therapies with evidence (e.g., ginkgo biloba)
    - Relevant organizations (e.g., Consortium of Multiple Sclerosis Centers)
    - Terms that are key to the target population (e.g., multiple sclerosis, inflammatory disease)

    NOTE: We switched some terms from one tier to another tier (National Multiple Sclerosis Society, therapies with evidence) so that we would have a pertinent term (National Multiple Sclerosis Society) and all therapies with evidence or Level U in the initial launch

  - Tier 2: terms with moderately focused applicability to the population of interest

    - Terms that weren't deemed overbroad (e.g., transcendental meditation)
    - Therapies with Level U findings (e.g., hyperbaric oxygen)
  - Tier 3: terms with least applicability
    - Therapies not studied in the guideline (e.g., dental amalgam replacement)
    - With least relevance of the set of terms (e.g., food allergies)
    - Deemed potentially overbroad (e.g., vegetarianism)
- Tested the potential reach of each tier of terms
  - Tier 2: had the narrowest reach but the greatest applicability
  - Tier 3: had the broadest reach but the narrowest applicability
  - Tier 1: had the most moderate degree of reach but a secondary level of applicability
- Determined that tier 1 (the tier with the midrange degree of both reach and applicability) would be used in the initial ad build, that tier 2 terms would be added next depending on the ad's performance (i.e., to widen the audience if need be), and that tier 3 terms would be added if needed later in the life of the ad

Category	Interest	Tier, action, rationale
Organization		
	AAN, American Academy of Neurology	Tier 2 – keep (org related to population of interest)
	FDA, Food and Drug Administration	Tier 1 – drop if number of followers too high (tangentially related org)
	American Holistic Nurses Association	Tier 2 – keep (org related to population of interest)
	American Osteopathic Association	Tier 2 – keep (org related to population of interest)
	MS Foundation	Tier 2 – keep (org related to population of interest)
	Multiple Sclerosis International Federation	Tier 2 – keep (org related to population of interest)
	Multiple Sclerosis Society of Canada	Tier 2 – keep (org related to population of interest)
	National Center for Complementary and Alternative Medicine	Tier 2 – keep (org related to population of interest)
	National Institute on Disability and Rehabilitation Research	Tier 2 – keep (org related to population of interest)
	National Multiple Sclerosis Society	Tier 1 – keep (org related to population of interest)
	Reflexology Association of America	Tier 2 – keep (org related to population of interest)
Health science		
	medical guidelines	Tier 1 – drop if number of followers too high (unknown whether many patients read guidelines)
	randomized, controlled trials	Tier 1 – drop if number of followers too high (unknown whether many patients aware of RCTs)
General disease-related topics		
	central nervous system, nervous system	Tier 1 – drop if number of followers too high (unknown whether many patients interested in CNS)
	immune system	Tier 1 – drop if number of followers too high (unknown whether many patients interested in immune system)
	immunology	Tier 1 – drop if number of followers too high (unknown whether many patients interested in immunology--more science-y)

	inflammation	Tier 1 – drop if number of followers too high (maybe too broad)
	inflammatory disease	Tier 2 – keep (key to target population)
	MS, multiple sclerosis	Tier 2 – keep (key to target population)
	neurology	Tier 1 – drop if number of followers too high (unknown whether many patients are interested in neurology in general)
	PPMS, primary progressive MS	Tier 1 – drop if number of followers too high (likely covered under
	RRMS, relapsing-remitting MS	Tier 1 – drop if number of followers too high (likely covered under
	SPMS, secondary progressive MS	Tier 1 – drop if number of followers too high (likely covered under
Signs/symptoms		
	brain lesions	Tier 1 – drop if number of followers too high (might have more patients with brain injury or cancer interested in this)
	bladder control	Tier 1 – drop if number of followers too high (too broad)
	cognitive problems	Tier 1 – drop if number of followers too high (might have more patients/families of patients with brain injury, dementia, or intellectual disability interested in this)
	depression, anxiety, mood problems	Tier 1 – drop if number of followers too high (too broad)
	digestive problems, constipation, diarrhea, nausea, vomiting	Tier 1 – drop if number of followers too high
	fatigue	Tier 1 – drop if number of followers too high (too broad)
	mobility problems, balance problems	Tier 1 – drop if number of followers too high (or keep as tier 1)
	pain, nerve pain, neuropathic pain, central pain	Tier 1 – drop if number of followers too high (or keep as tier 1)
	paresthesia	Tier 1 – drop if number of followers too high (too broad)
	muscle weakness, muscle problems	Tier 1 – drop if number of followers too high (or keep as tier 1)
	respiratory problems, breathing problems	Tier 1 – drop if number of followers too high (too broad)
	sleep problems, sleep	Tier 1 – drop if number of followers too high (too broad)

	spasticity, spasticity symptoms, muscle cramps/spasms, muscle stiffness	Tier 1 – drop if number of followers too high (or keep as tier 1)
	tremor	Tier 1 – drop if number of followers too high (might have more people with movement disorders indicating this as an interest)
	vision problems, vision	Tier 1 – drop if number of followers too high (too broad)
General CAM-related topics		
	alternative medicine, alternative health	Tier 2 – keep (key to target population)
	Andrew Weil, MD; Weil Foundation	Tier 1 – drop if number of followers too high (an MD and holistic medicine advocate--related but not key interest)
	Ayurvedic medicine	Tier 1 – drop if number of followers too high (related to Padma 28 but not key interest)
	Buddhism	Tier 1 – drop if number of followers too high (related but not key interest)
	Byron Katie	Tier 1 – drop if number of followers too high (an advocate for a quasi-cognitive behavioral therapy and quasi-mindfulness-based approach--related but not key interest)
	CAM, complementary and alternative medicine	Tier 2 – keep (key to target population)
	chakra healing, chakras	Tier 3 – drop first to lower numbers (tangentially related)
	complementary medicine	Tier 2 – keep (key to target population)
	Deepak Chopra	Tier 1 – drop if number of followers too high (an advocate for holistic medicine--related but not key interest)
	Eckhart Tolle	Tier 1 – drop if number of followers too high (an advocate for a quasi-cognitive behavioral therapy and quasi-mindfulness-based approach--related but not key interest)
	food sensitivities, food allergies	Tier 3 – drop first to lower numbers (tangentially related)
	functional neurology	Tier 3 – drop first to lower numbers (tangentially related)

	Henry Emmons, MD	Tier 1 – drop if number of followers too high (an advocate for a holistic approach to psychiatry that incorporates mindfulness--related but not key interest)
	holistic dentistry	Tier 3 – drop first to lower numbers (tangentially related)
	herbal medicine, herbal therapy	Tier 2 – keep (key to target population)
	holistic medicine	Tier 1 – drop if number of followers too high (related but not key interest)
	integrative medicine	Tier 1 – drop if number of followers too high (related but not key interest)
	Jon Kabat-Zinn, PhD	Tier 1 – drop if number of followers too high (established mindfulness-based stress reduction approach to managing physical and psychological illness and chronic pain--related but not key interest)
	Louise Hay	Tier 1 – drop if number of followers too high (an advocate of positive thinking for treating health--related but not key interest)
	Marianne Williamson	Tier 1 – drop if number of followers too high (an advocate for affirmations as a method of dealing with emotional problems--related but not key interest)
	Mehmet Oz, MD	Tier 1 – drop if number of followers too high (Dr. Oz of TV--related but not key interest; also maybe too broad)
	new age	Tier 1 – drop if number of followers too high (too broad)
	Sanjay Gupta, MD	Tier 1 – drop if number of followers too high (Dr. Gupta of CNN--related but not key interest; also maybe too broad)
	stress management	Tier 1 – drop if number of followers too high (too broad)
	transcendental meditation	Tier 1 – drop if number of followers too high (related but not key interest)
	veganism	Tier 3 – drop first to lower numbers (tangentially related)
	vegetarianism	Tier 3 – drop first to lower numbers (tangentially related)
	Wayne Dyer, MD	Tier 1 – drop if number of followers too high (Dr. Dyer of public TV and DVDs about improving health through positive thinking and self-care--related but not key interest; also maybe too broad)

	wellness	Tier 1 – drop if number of followers too high (too broad)
	Zen meditation	Tier 1 – drop if number of followers too high (related but not key interest)
Therapies - General		
	chelation therapy***	Tier 3 – drop first to lower numbers (not studied in the CPG)
	disease-modifying therapies, disease modifying therapies, DMTs**	Tier 1 – drop if number of followers too high (Level U)--or go with tier 1 because it's an important treatment in MS
	Chinese medicine, traditional Chinese medicine, oriental medicine***	Tier 1 – drop if number of followers too high (not studied in the CPG)--or go with tier 1 because it's relevant and involves herbs
Therapies - Dietary supplements		
	acetyl-L-carnitine, ALCAR**	Tier 1 – drop if number of followers too high (Level U)
	carnitine***	Tier 3 – drop first to lower numbers (not studied in the CPG)
	creatine, creatine monohydrate**	Tier 1 – drop if number of followers too high (Level U)
	glucosamine, glucosamine sulfate**	Tier 1 – drop if number of followers too high (Level U)
	inosine**	Tier 1 – drop if number of followers too high (Level U)
	Cari Loder regimen*	Tier 1 – keep (evidence)
	low-dose naltrexone, LDN**	Tier 1 – drop if number of followers too high (Level U)
	omega-3 fatty acids, fish oil,* low-fat diet	Tier 1 – keep (evidence)
	threonine**	Tier 1 – drop if number of followers too high (Level U)
Therapies - Energy medicine		
	magnetic therapy*	Tier 1 – keep (evidence)

	naturopathic medicine**	Tier 1 – drop if number of followers too high (Level U)
	neural therapy**	Tier 1 – drop if number of followers too high (Level U)
Therapies – Herbs		
	cannabis*	Tier 1 – keep (evidence)
	cannabinoids*	Tier 1 – keep (evidence)
	CBD, cannabidiol*	Tier 1 – keep (evidence)
	marijuana*	Tier 1 – keep (evidence)
	Marinol, dronabinol*	Tier 1 – keep (evidence)
	medical marijuana*	Tier 1 – keep (evidence)
	OCE, oral cannabis extract*	Tier 1 – keep (evidence)
	Sativex oromucosal cannabinoid spray, nabiximols*	Tier 1 – keep (evidence)
	THC, tetrahydrocannabinol*	Tier 1 – keep (evidence)
	GB, ginkgo biloba*	Tier 1 – keep (evidence)
	Padma 28**	Tier 1 – drop if number of followers too high (Level U)
Therapies - Manipulative and body-based		
	acupuncture**	Tier 1 – drop if number of followers too high (Level U)
	chiropractic medicine, chiropractic***	Tier 3 – drop first to lower numbers (not studied in the CPG)
	hippotherapy, horseback riding, therapeutic horseback riding**	Tier 1 – drop if number of followers too high (Level U)
	massage, massage therapy**	Tier 1 – drop if number of followers too high (Level U)
	PMRT, progressive muscle relaxation, progressive muscle relaxation therapy**	Tier 1 – drop if number of followers too high (Level U)
	reflexology*	Tier 1 – keep (evidence)
	tai chi***	Tier 3 – drop first to lower numbers (not studied in the CPG)
	yoga**	Tier 1 – drop if number of followers too high (Level U)
Therapies - Mind-body medicine		
	biofeedback**	Tier 1 – drop if number of followers too high (Level U)

	hypnosis**	Tier 1 – drop if number of followers too high (Level U)
	mindfulness-based intervention**	Tier 1 – drop if number of followers too high (Level U)
	mindfulness, mindfulness training**	Tier 1 – drop if number of followers too high (Level U)
	music therapy**	Tier 1 – drop if number of followers too high (Level U)
	bee sting therapy, bee venom*	Tier 1 – keep (evidence)
	dental amalgam replacement***	Tier 3 – drop first to lower numbers (not studied in the CPG)
	hyperbaric oxygen**	Tier 1 – drop if number of followers too high (Level U)
	transdermal histamine with caffeine**	Tier 1 – drop if number of followers too high (Level U)

NOTE: The asterisks in this list indicate the following:

1 asterisk = therapy with recommendation

2 asterisks = therapy with Level U finding

3 asterisks = therapy mentioned in guideline as not having been studied in the guideline (studies for these were too poor in quality to be included in the analysis)
