# Supplementary material for: The Impact of Social Media on Dissemination and Implementation of Clinical Practice Guidelines: A Longitudinal Observational Study
Source: J Med Internet Res. 2015 Aug 13;17(8):e193. doi: 10.2196/jmir.4414 (PMC4736287; doi:10.2196/jmir.4414)
Supplement: Multimedia Appendix 2 [file jmir_v17i8e193_app2.pdf]

MULTIMEDIA APPENDIX 2: SURVEY DEVELOPMENT METHODS AND DATA COLLECTION PROCEDURE

Survey Development Methods:

Survey questions were written and formatted in accordance with the American Association of Public Opinion Research (AAPOR's) evidence-based, best practices for survey research. Surveys underwent cognitive testing with 10 volunteers (5 physicians and 5 patients), who were drawn from the population of interest and were ineligible for the subsequent study. They were asked to complete the surveys, describe what the questions and response options meant to them, identify questions and instructions that were difficult to understand (and suggest alternative wording). Participants were also asked if there were any questions that were repetitive or additional questions that should be asked. Responses were timed and participants' ability to easily complete the survey was assessed. Surveys were revised based on this testing.

Survey Data Collection Procedure: All participants were contacted by email and invited to participate in a "research project examining the effectiveness of different approaches to dissemination of an AAN guideline titled 'CAM in MS.'" They were informed that participation would entail completing a survey that would take them no more than 5 to 10 minutes and would be of no direct clinical benefit to them. A link to the online version of the survey was provided. Reminder email invitations were sent to non-respondents after one week. One week after the reminder emails, surveys were mailed in paper format to non-respondents. Clinician surveys were mailed in envelopes stamped "time-sensitive" in red ink,

to increase the likelihood that they would be opened and read. Two weeks after the mailed survey, a final reminder email was sent. Surveys closed one week later.

All survey invitations contained contact information that participants could use to decline study participation. Clinicians who completed the study were entered into a drawing to win one of the following prizes: (a) complimentary registration in AAN’s practice management webinar 2014 series; (b) complimentary registration to either the next AAN Fall Conference or the next AAN Annual Meeting; or (c) one-year free subscription to *Continuum*®, AAN’s official continuing medical education and self-assessment journal. Patients who completed the study were entered into a drawing to win $50.
