# Supplementary material for: The Impact of Social Media on Dissemination and Implementation of Clinical Practice Guidelines: A Longitudinal Observational Study
Source: J Med Internet Res. 2015 Aug 13;17(8):e193. doi: 10.2196/jmir.4414 (PMC4736287; doi:10.2196/jmir.4414)
Supplement: Multimedia Appendix 3 [file jmir_v17i8e193_app3.pdf]

Patient Pre-Dissemination Survey

Use of Complementary and Alternative Treatments in Multiple Sclerosis

This survey asks about your attitudes regarding complementary and alternative medicine (CAM) treatments for multiple sclerosis (MS), how you get your information about MS, and how you think your MS should be treated. This survey should take no more than 5–10 minutes to complete. We appreciate your time and feedback.

Unless otherwise instructed, please select one (1) response that best reflects your answer.
There are no right or wrong answers.

If you are a caregiver, please fill out the survey *as if you are the patient.*
☐ **Check here if this survey was completed by a patient representative or caregiver.**

For questions, please contact:

Pushpa Narayanaswami, MBBS, DM, FAAN
Neurology TCC-8, BIDMC
330 Brookline Avenue
Boston, MA 02215
Phone: (617)-667-8130
Fax: (617)-667-3175
Email: pnarayan@bidmc.harvard.edu

Before we begin, we have a few questions about your use of the Internet.

1. How often are you on the Internet?
   - ☐ I rarely use a computer or the Internet
   - ☐ I use the Internet once a week
   - ☐ I use the Internet several times per week
   - ☐ I use the Internet every day

2. Do you have ***any*** disability that makes it hard for you to use the Internet?
   - ☐ Yes
   - ☐ No

3. If you use the Internet, what is your favorite site to go to learn about MS? ______________________

4. Please name other sites you visit for MS information:

   ____

   ____

   ____

   ____

   ____

   ____

The National Center for Complementary and Alternative Medicine (NCCAM) defines complementary and alternative medicine (CAM) as medical practices and products that are not generally considered part of conventional medicine.

- ***Conventional medicine*** is medicine practiced by medical doctors and health professionals such as physical therapists, psychologists, and registered nurses. Examples of conventional medicine are vaccines, prescriptions, and surgery.
- ***Complementary medicine*** is non-conventional medicine practiced **together with** conventional medicine. An example is using acupuncture **in addition to** medication to help lessen pain.
- ***Alternative medicine*** refers to use of non-conventional medicine practiced **instead of** conventional medicine. An example of "non-conventional" therapy is using an herbal supplement such as ginkgo biloba for memory.

5. Have you ***heard of*** CAM therapies before?
   - ☐ Yes
   - ☐ No

Below is a list of CAM therapies. For each therapy listed below, please tell us whether you have ***ever used*** the therapy and whether the therapy ***helped to ease*** your symptoms.

6. For each therapy below, please answer each question.

	Have you ***ever used*** this therapy?		***If you used it***, did this therapy help ease your symptoms?	
	Yes	No	Yes	No
Acupuncture	☐	☐	☐	☐
Amalgam replacement	☐	☐	☐	☐
Bee venom (sting)	☐	☐	☐	☐
Biofeedback	☐	☐	☐	☐
Cannabis extract (e.g., Cesamet, Marinol)	☐	☐	☐	☐
Carnitine	☐	☐	☐	☐
Chelation	☐	☐	☐	☐
Chiropractic care	☐	☐	☐	☐
Creatine	☐	☐	☐	☐
Ginkgo biloba	☐	☐	☐	☐
Glucosamine	☐	☐	☐	☐
Hippotherapy (therapeutic horseback riding)	☐	☐	☐	☐
Hyperbaric oxygen	☐	☐	☐	☐
Inosine	☐	☐	☐	☐
Linoleic acid	☐	☐	☐	☐
Lofepramine	☐	☐	☐	☐
Low-dose naltrexone	☐	☐	☐	☐
Magnetic therapy	☐	☐	☐	☐
Massage therapy	☐	☐	☐	☐
Mindfulness	☐	☐	☐	☐
Music therapy	☐	☐	☐	☐
Naturopathic medicine	☐	☐	☐	☐
Neural therapy	☐	☐	☐	☐
Omega-3 fatty acid supplementation (e.g., fish oil)	☐	☐	☐	☐

Please continue to tell us whether you ***have ever used*** these CAM therapies and ***if you used it***, whether it ***helped to ease*** your symptoms.

	Have you ***ever used*** this therapy?		***If you used it***, did this therapy help ease your symptoms?	
	Yes	No	Yes	No
Padma 28	☐	☐	☐	☐
Phenylalanine	☐	☐	☐	☐
Progressive muscle relaxation	☐	☐	☐	☐
Reflexology	☐	☐	☐	☐
Smoking marijuana	☐	☐	☐	☐
Tai chi	☐	☐	☐	☐
Threonine	☐	☐	☐	☐
Transdermal histamine (e.g., Prokarin patch)	☐	☐	☐	☐
Yoga	☐	☐	☐	☐
Other (please describe): ____________________ ____________________ ____________________	☐	☐	☐	☐

If you have *never* used any of these therapies, PLEASE GO TO QUESTION 9, PAGE 7.

7. Think about the therapies you selected in Question 6 (above). Did you ***start using*** any of them within the ***last 6 months****?* ***Please check all that apply****.*

   I ***started using*** this therapy within the ***last 6 months:***

   - ☐ Acupuncture
   - ☐ Amalgam replacement
   - ☐ Bee venom (sting)
   - ☐ Biofeedback
   - ☐ Cannabis extract (e.g., Cesamet, Marinol)
   - ☐ Carnitine
   - ☐ Chelation
   - ☐ Chiropractic care
   - ☐ Creatine
   - ☐ Ginkgo biloba
   - ☐ Glucosamine
   - ☐ Hippotherapy (therapeutic horseback riding)
   - ☐ Hyperbaric oxygen
   - ☐ Inosine
   - ☐ Linoleic acid
   - ☐ Lofepramine
   - ☐ Low-dose naltrexone
   - ☐ Magnetic therapy
   - ☐ Massage therapy
   - ☐ Mindfulness
   - ☐ Music therapy
   - ☐ Naturopathic medicine
   - ☐ Neural therapy
   - ☐ Omega-3 fatty acid supplementation (e.g., fish oil)
   - ☐ Padma 28
   - ☐ Phenylalanine
   - ☐ Progressive muscle relaxation
   - ☐ Reflexology
   - ☐ Smoking marijuana
   - ☐ Tai chi
   - ☐ Threonine
   - ☐ Transdermal histamine (e.g., Prokarin patch)
   - ☐ Yoga
   - ☐ Other (please describe):
     ____
     ____
     ____
   - ☐ ***I did not start any of these in the last 6 months***

We know that there are many different reasons why people ***start using*** CAM therapies. We would like to know why you started using the therapy (or therapies) you selected above.

If you started using more than one (1) CAM therapy, please ***check all the reasons that apply***. Even though you may have different reasons for ***starting*** the use of different CAM therapies, we only need to know what your reasons are overall.

For ***all*** the therapies you ***started*** in the last 6 months:

8. What were your overall reasons for choosing ***to start*** this therapy (or therapies)?
   Please check all that apply.
   - ☐ ***I did not start any of these CAM therapies in the last 6 months***
   - ☐ I hoped it would improve/help manage my MS symptoms
   - ☐ It was recommended by a friend or family member
   - ☐ It was recommended by a physician/care provider
   - ☐ I learned about it from TV
   - ☐ I learned about it from the Internet
   - ☐ I read a clinical practice guideline
   - ☐ My insurance now pays for it
   - ☐ It has recently become available in my area
   - ☐ It has recently become legal in my area
   - ☐ Other (please describe):____

The next set of questions is about specific CAM therapies. **Please select one (1) response that best reflects the extent to which you agree or disagree with each of the statements below.**

	**Agree**	**Somewhat agree**	**Somewhat disagree**	**Disagree**	**Not sure**
9. Magnetic therapy is effective for treating fatigue in people with MS.	☐	☐	☐	☐	☐
10. Taking oral cannabis extract is effective for treating tremors in people with MS.	☐	☐	☐	☐	☐
11. Smoking marijuana (cannabis) is effective for treating balance, posture, or pain in people with MS.	☐	☐	☐	☐	☐
12. Taking ginkgo biloba orally is effective for improving memory in people with MS.	☐	☐	☐	☐	☐
13. Hyperbaric oxygen is effective for improving symptoms in people with MS.	☐	☐	☐	☐	☐
14. Bee sting therapy is effective for reducing MRI lesions in people with MS.	☐	☐	☐	☐	☐

The next set of questions asks about your ***intentions*** to use CAM therapies.

15. Do you ***plan to initiate discussions about*** any of these CAM therapies for MS with your doctor who treats your MS? ***Please check all that apply*** *or select "No" if you do not plan to initiate any discussions about any CAM therapies with your doctor.*

- ☐ ***No, I do not plan to initiate any discussions about any CAM therapies with my doctor who treats my MS.***

- ☐ Acupuncture
- ☐ Amalgam replacement
- ☐ Bee venom (sting)
- ☐ Biofeedback
- ☐ Cannabis extract (e.g., Cesamet, Marinol)
- ☐ Carnitine
- ☐ Chelation
- ☐ Chiropractic care
- ☐ Creatine
- ☐ Ginkgo biloba
- ☐ Glucosamine
- ☐ Hippotherapy (therapeutic horseback riding)
- ☐ Hyperbaric oxygen
- ☐ Inosine
- ☐ Linoleic acid
- ☐ Lofepramine
- ☐ Low-dose naltrexone
- ☐ Magnetic therapy
- ☐ Massage therapy
- ☐ Mindfulness
- ☐ Music therapy
- ☐ Naturopathic medicine
- ☐ Neural therapy
- ☐ Omega-3 fatty acid supplementation (e.g., fish oil)
- ☐ Padma 28
- ☐ Phenylalanine
- ☐ Progressive muscle relaxation
- ☐ Reflexology
- ☐ Smoking marijuana
- ☐ Tai chi
- ☐ Threonine
- ☐ Transdermal histamine (e.g., Prokarin patch)
- ☐ Yoga
- ☐ Other (please describe): ____________________________________ ____________________________________ ____________________________________

16. Do you ***intend to start using*** any CAM therapies for treatment of your MS?
    - ☐ Yes
    - ☐ No

17. Do you ***intend to stop using*** any CAM therapies for treatment of your MS?
    - ☐ Yes
    - ☐ No

If you said "No" to BOTH 16 and 17, PLEASE GO TO QUESTION 19, PAGE 10.

18. Which CAM therapies do you ***intend to start using*** or ***stop using*** for treatment of your MS?

	I intend to ***start using*** this CAM therapy.		I intend to ***stop using*** this CAM therapy.	
	Yes	No	Yes	No
Acupuncture	☐	☐	☐	☐
Amalgam replacement	☐	☐	☐	☐
Bee venom (sting)	☐	☐	☐	☐
Biofeedback	☐	☐	☐	☐
Cannabis extract (e.g., Cesamet, Marinol)	☐	☐	☐	☐
Carnitine	☐	☐	☐	☐
Chelation	☐	☐	☐	☐
Chiropractic care	☐	☐	☐	☐
Creatine	☐	☐	☐	☐
Ginkgo biloba	☐	☐	☐	☐
Glucosamine	☐	☐	☐	☐
Hippotherapy (therapeutic horseback riding)	☐	☐	☐	☐
Hyperbaric oxygen	☐	☐	☐	☐
Inosine	☐	☐	☐	☐
Linoleic acid	☐	☐	☐	☐
Lofepramine	☐	☐	☐	☐
Low-dose naltrexone	☐	☐	☐	☐
Magnetic therapy	☐	☐	☐	☐
Massage therapy	☐	☐	☐	☐
Mindfulness	☐	☐	☐	☐
Music therapy	☐	☐	☐	☐
Naturopathic medicine	☐	☐	☐	☐
Neural therapy	☐	☐	☐	☐
Omega-3 fatty acid supplementation (e.g., fish oil)	☐	☐	☐	☐
Padma 28	☐	☐	☐	☐
Phenylalanine	☐	☐	☐	☐
Progressive muscle relaxation	☐	☐	☐	☐
Reflexology	☐	☐	☐	☐
Smoking marijuana	☐	☐	☐	☐
Tai chi	☐	☐	☐	☐
Threonine	☐	☐	☐	☐
Transdermal histamine (e.g., Prokarin patch)	☐	☐	☐	☐
Yoga	☐	☐	☐	☐
Other (please describe): ____________________________ ____________________________ ____________________________	☐	☐	☐	☐

A **clinical practice guideline** is a document that helps health care providers make decisions. Guidelines look at all the ways to diagnose and treat a disease, and this helps health care providers make a good decision for a patient. Guidelines from the AAN are developed by neurologists and other experts. The guidelines are based on a careful review of all available scientific studies for a specific brain disease.

19. Did you know that the AAN has developed a guideline about the use of CAM therapies in MS?
    - ☐ Yes
    - ☐ No

The final set of questions is about you.

20. How long has it been since you were diagnosed with MS?
    - ☐ Less than 1 year
    - ☐ 1 – 5 years
    - ☐ 6 – 10 years
    - ☐ 11 – 20 years
    - ☐ More than 20 years

21. Are you able to walk?
    - ☐ I am walking without assistance
    - ☐ I am walking with assistance
    - ☐ I cannot walk; I rely on a wheelchair

22. Do you have problems with your vision?
    - ☐ I have no problems with my vision
    - ☐ I have some visual impairment
    - ☐ I have severe visual impairment/blindness

23. Do you have difficulty using your hands?
    - ☐ I have no difficulty using my hands
    - ☐ I have some difficulty using my hands
    - ☐ I have severe difficulty using my hands
    - ☐ I cannot use my hands

24. How old are you?
    ________ years old

25. What is your gender?
    - ☐ Female
    - ☐ Male
    - ☐ Prefer not to answer

26. Do you consider yourself Hispanic or Latino/a?
    - ☐ Yes, Hispanic or Latino/a
    - ☐ No

27. What is your race? ***Please check all that apply.***
    - ☐ American Indian or Native American
    - ☐ Asian
    - ☐ Black or African American
    - ☐ Hawaiian or other Pacific Islander
    - ☐ White
    - ☐ Other (please specify): ______________________________

28. What is your highest level of education?
    - ☐ Did not attend high school
    - ☐ Some high school education
    - ☐ High school diploma
    - ☐ Some college education
    - ☐ College diploma
    - ☐ Some postgraduate education
    - ☐ Postgraduate diploma

Thank you!

Please return your survey in the self-addressed return envelope.

Patient Post-Dissemination Survey 1

AMERICAN ACADEMY OF NEUROLOGY INSTITUTE

Use of Complementary and Alternative Treatments in Multiple Sclerosis

This survey asks about your attitudes regarding complementary and alternative medicine (CAM) treatments for multiple sclerosis (MS), how you get your information about MS, and how you think your MS should be treated. This survey should take no more than 5–10 minutes to complete. We appreciate your time and feedback.

Unless otherwise instructed, please select one (1) response that best reflects your answer. **There are no right or wrong answers.**

If you are a caregiver, please fill out the survey *as if you are the patient.*
☐ **Check here if this survey was completed by a patient representative or caregiver.**

For questions, please contact:
Pushpa Narayanaswami, MBBS, DM, FAAN
Neurology TCC-8, BIDMC
330 Brookline Avenue
Boston, MA 02215
Phone: (617) 667-8130
Fax: (617) 667-3175
Email: pnarayan@bidmc.harvard.edu

Before we begin, we have a few questions about your use of the Internet.

1. How often are you on the Internet?
   - ☐ I rarely use a computer or the Internet
   - ☐ I use the Internet once a week
   - ☐ I use the Internet several times per week
   - ☐ I use the Internet every day

2. Do you have ***any*** disability that makes it hard for you to use the Internet?
   - ☐ Yes
   - ☐ No

3. If you use the Internet, what is your favorite site to go to learn about MS? ______________________

4. Please name other sites you visit for MS information:

   ____

   ____

   ____

   ____

   ____

   ____

The National Center for Complementary and Alternative Medicine (NCCAM) defines complementary and alternative medicine (CAM) as medical practices and products that are not generally considered part of conventional medicine.

- ***Conventional medicine*** is medicine practiced by medical doctors and health professionals such as physical therapists, psychologists, and registered nurses. Examples of conventional medicine are vaccines, prescriptions, and surgery.
- ***Complementary medicine*** is non-conventional medicine practiced **together with** conventional medicine. An example is using acupuncture **in addition to** medication to help lessen pain.
- ***Alternative medicine*** refers to use of non-conventional medicine practiced **instead of** conventional medicine. An example of "non-conventional" therapy is using an herbal supplement such as ginkgo biloba for memory.

5. Have you ***heard of*** CAM therapies before?
   - ☐ Yes
   - ☐ No

Below is a list of CAM therapies. For each therapy listed below, please tell us whether you have ***ever used*** the therapy and whether the therapy ***helped to ease*** your symptoms.

6. For each therapy below, please answer each question.

	Have you ***ever used*** this therapy?		***If you used it***, did this therapy help ease your symptoms?	
	Yes	No	Yes	No
Acupuncture	☐	☐	☐	☐
Amalgam replacement	☐	☐	☐	☐
Bee venom (sting)	☐	☐	☐	☐
Biofeedback	☐	☐	☐	☐
Cannabis extract (e.g., Cesamet, Marinol)	☐	☐	☐	☐
Carnitine	☐	☐	☐	☐
Chelation	☐	☐	☐	☐
Chiropractic care	☐	☐	☐	☐
Creatine	☐	☐	☐	☐
Ginkgo biloba	☐	☐	☐	☐
Glucosamine	☐	☐	☐	☐
Hippotherapy (therapeutic horseback riding)	☐	☐	☐	☐
Hyperbaric oxygen	☐	☐	☐	☐
Inosine	☐	☐	☐	☐
Linoleic acid	☐	☐	☐	☐
Lofepramine	☐	☐	☐	☐
Low-dose naltrexone	☐	☐	☐	☐
Magnetic therapy	☐	☐	☐	☐
Massage therapy	☐	☐	☐	☐
Mindfulness	☐	☐	☐	☐
Music therapy	☐	☐	☐	☐
Naturopathic medicine	☐	☐	☐	☐
Neural therapy	☐	☐	☐	☐
Omega-3 fatty acid supplementation (e.g., fish oil)	☐	☐	☐	☐

Please continue to tell us whether ***you have ever used*** these CAM therapies and ***if you used it***, whether it ***helped to ease*** your symptoms.

	Have you ***ever used*** this therapy?		***If you used it***, did this therapy help ease your symptoms?	
	Yes	No	Yes	No
Padma 28	☐	☐	☐	☐
Phenylalanine	☐	☐	☐	☐
Progressive muscle relaxation	☐	☐	☐	☐
Reflexology	☐	☐	☐	☐
Smoking marijuana	☐	☐	☐	☐
Tai chi	☐	☐	☐	☐
Threonine	☐	☐	☐	☐
Transdermal histamine (e.g., Prokarin patch)	☐	☐	☐	☐
Yoga	☐	☐	☐	☐
Other (please describe): ____________________ ____________________ ____________________	☐	☐	☐	☐

The next set of questions is about specific CAM therapies. **Please select one (1) response that best reflects the extent to which you agree or disagree with each of the statements below.**

	**Agree**	**Somewhat agree**	**Somewhat disagree**	**Disagree**	**Not sure**
7. Magnetic therapy is effective for treating fatigue in people with MS.	☐	☐	☐	☐	☐
8. Taking oral cannabis extract is effective for treating tremors in people with MS.	☐	☐	☐	☐	☐
9. Smoking marijuana (cannabis) is effective for treating balance, posture, or pain in people with MS.	☐	☐	☐	☐	☐
10. Taking ginkgo biloba orally is effective for improving memory in people with MS.	☐	☐	☐	☐	☐
11. Hyperbaric oxygen is effective for improving symptoms in people with MS.	☐	☐	☐	☐	☐
12. Bee sting therapy is effective for reducing MRI lesions in people with MS.	☐	☐	☐	☐	☐

The next set of questions asks about your ***intentions*** to use CAM therapies.

13. Do you ***plan to initiate discussions*** *about* any of these CAM therapies for MS with your doctor who treats your MS? ***Please check all that apply*** *or select "No" if you do not plan to initiate any discussions about any CAM therapies with your doctor.*

□ ***No, I do not plan to initiate any discussions about any CAM therapies with my doctor who treats my MS.***

☐ Acupuncture
☐ Amalgam replacement
☐ Bee venom (sting)
☐ Biofeedback
☐ Cannabis extract (e.g., Cesamet, Marinol)
☐ Carnitine
☐ Chelation
☐ Chiropractic care
☐ Creatine
☐ Ginkgo biloba
☐ Glucosamine
☐ Hippotherapy (therapeutic horseback riding)
☐ Hyperbaric oxygen
☐ Inosine
☐ Linoleic acid
☐ Lofepramine
☐ Low-dose naltrexone
☐ Magnetic therapy
☐ Massage therapy
☐ Mindfulness
☐ Music therapy
☐ Naturopathic medicine
☐ Neural therapy
☐ Omega-3 fatty acid supplementation (e.g., fish oil)
☐ Padma 28
☐ Phenylalanine
☐ Progressive muscle relaxation
☐ Reflexology
☐ Smoking marijuana
☐ Tai chi
☐ Threonine
☐ Transdermal histamine (e.g., Prokarin patch)
☐ Yoga
☐ Other (please describe): ____________________________________
____
____

14. Do you ***intend to start using*** any CAM therapies for treatment of your MS?
☐ Yes
☐ No

15. Do you ***intend to stop using*** any CAM therapies for treatment of your MS?
☐ Yes
☐ No

If you said "No" to BOTH 14 and 15, GO TO QUESTION 17, PAGE 8.

16. Which CAM therapies do you ***intend to start using*** or ***stop using*** for treatment of your MS?

	I intend to ***start using*** this CAM therapy.		I intend to ***stop using*** this CAM therapy.	
	Yes	No	Yes	No
Acupuncture	☐	☐	☐	☐
Amalgam replacement	☐	☐	☐	☐
Bee venom (sting)	☐	☐	☐	☐
Biofeedback	☐	☐	☐	☐
Cannabis extract (e.g., Cesamet, Marinol)	☐	☐	☐	☐
Carnitine	☐	☐	☐	☐
Chelation	☐	☐	☐	☐
Chiropractic care	☐	☐	☐	☐
Creatine	☐	☐	☐	☐
Ginkgo biloba	☐	☐	☐	☐
Glucosamine	☐	☐	☐	☐
Hippotherapy (therapeutic horseback riding)	☐	☐	☐	☐
Hyperbaric oxygen	☐	☐	☐	☐
Inosine	☐	☐	☐	☐
Linoleic acid	☐	☐	☐	☐
Lofepramine	☐	☐	☐	☐
Low-dose naltrexone	☐	☐	☐	☐
Magnetic therapy	☐	☐	☐	☐
Massage therapy	☐	☐	☐	☐
Mindfulness	☐	☐	☐	☐
Music therapy	☐	☐	☐	☐
Naturopathic medicine	☐	☐	☐	☐
Neural therapy	☐	☐	☐	☐
Omega-3 fatty acid supplementation (e.g., fish oil)	☐	☐	☐	☐
Padma 28	☐	☐	☐	☐
Phenylalanine	☐	☐	☐	☐
Progressive muscle relaxation	☐	☐	☐	☐
Reflexology	☐	☐	☐	☐
Smoking marijuana	☐	☐	☐	☐
Tai chi	☐	☐	☐	☐
Threonine	☐	☐	☐	☐
Transdermal histamine (e.g., Prokarin patch)	☐	☐	☐	☐
Yoga	☐	☐	☐	☐
Other (please describe): ____________________ ____________________ ____________________	☐	☐	☐	☐

The next set of questions is about your ***actual behavior*** in relation to CAM therapies.

17. Since *March 2014*, have you **discussed** any of these CAM therapies for MS with your doctor who treats your MS? ***Please check all that apply*** *or select "No" if you have not discussed any CAM therapies with your doctor.*

- ☐ ***No, I have not discussed any CAM therapies with my doctor who treats my MS.***

- ☐ Acupuncture
- ☐ Amalgam replacement
- ☐ Bee venom (sting)
- ☐ Biofeedback
- ☐ Cannabis extract (e.g., Cesamet, Marinol)
- ☐ Carnitine
- ☐ Chelation
- ☐ Chiropractic care
- ☐ Creatine
- ☐ Ginkgo biloba
- ☐ Glucosamine
- ☐ Hippotherapy (therapeutic horseback riding)
- ☐ Hyperbaric oxygen
- ☐ Inosine
- ☐ Linoleic acid
- ☐ Lofepramine
- ☐ Low-dose naltrexone
- ☐ Magnetic therapy
- ☐ Massage therapy
- ☐ Mindfulness
- ☐ Music therapy
- ☐ Naturopathic medicine
- ☐ Neural therapy
- ☐ Omega-3 fatty acid supplementation (e.g., fish oil)
- ☐ Padma 28
- ☐ Phenylalanine
- ☐ Progressive muscle relaxation
- ☐ Reflexology
- ☐ Smoking marijuana
- ☐ Tai chi
- ☐ Threonine
- ☐ Transdermal histamine (e.g., Prokarin patch)
- ☐ Yoga
- ☐ Other (please describe): ____________________________________
____
____

18. Since *March 2014*, have you ***started using*** or ***stopped using*** any of these CAM therapies for MS?

	I have ***started using*** this CAM therapy since *March 2014*		I have ***stopped using*** this CAM therapy since *March 2014*	
	Yes	No	Yes	No
Acupuncture	☐	☐	☐	☐
Amalgam replacement	☐	☐	☐	☐
Bee venom (sting)	☐	☐	☐	☐
Biofeedback	☐	☐	☐	☐
Cannabis extract (e.g., Cesamet, Marinol)	☐	☐	☐	☐
Carnitine	☐	☐	☐	☐
Chelation	☐	☐	☐	☐
Chiropractic care	☐	☐	☐	☐
Creatine	☐	☐	☐	☐
Ginkgo biloba	☐	☐	☐	☐
Glucosamine	☐	☐	☐	☐
Hippotherapy (therapeutic horseback riding)	☐	☐	☐	☐
Hyperbaric oxygen	☐	☐	☐	☐
Inosine	☐	☐	☐	☐
Linoleic acid	☐	☐	☐	☐
Lofepramine	☐	☐	☐	☐
Low-dose naltrexone	☐	☐	☐	☐
Magnetic therapy	☐	☐	☐	☐
Massage therapy	☐	☐	☐	☐
Mindfulness	☐	☐	☐	☐
Music therapy	☐	☐	☐	☐
Naturopathic medicine	☐	☐	☐	☐
Neural therapy	☐	☐	☐	☐
Omega-3 fatty acid supplementation (e.g., fish oil)	☐	☐	☐	☐
Padma 28	☐	☐	☐	☐
Phenylalanine	☐	☐	☐	☐
Progressive muscle relaxation	☐	☐	☐	☐
Reflexology	☐	☐	☐	☐
Smoking marijuana	☐	☐	☐	☐
Tai chi	☐	☐	☐	☐
Threonine	☐	☐	☐	☐
Transdermal histamine (e.g., Prokarin patch)	☐	☐	☐	☐
Yoga	☐	☐	☐	☐
Other (please describe): ____________________________ ____________________________ ____________________________	☐	☐	☐	☐

We know that there are many different reasons why people ***start or stop using*** CAM therapies. We would like to know why you ***started*** or ***stopped*** using the therapy (or therapies) you selected previously.

If you started or stopped using more than one (1) CAM therapy, please ***check all the reasons that apply***. Even though you may have different reasons for *starting or stopping* the use of different CAM therapies, we only need to know what your reasons are overall.

19. For all the therapies you ***started*** since *March 2014*:

    What were your overall reasons for choosing ***to start*** this therapy (or therapies)?
    Please check all that apply.
    ☐ ***I have not started any CAM therapies since March 2014***
    ☐ I hoped it would improve/help manage my MS symptoms
    ☐ It was recommended by a friend or family member
    ☐ It was recommended by a physician/care provider
    ☐ I learned about it from TV
    ☐ I learned about it from the Internet
    ☐ I read a clinical practice guideline
    ☐ My insurance now pays for it
    ☐ It has recently become available in my area
    ☐ It has recently become legal in my area
    ☐ Other (please describe):________________________

20. For all the therapies you ***stopped*** since *March 2014*:

    What were your overall reasons for choosing ***to stop*** this therapy (or therapies)?
    Please check all that apply.
    ☐ ***I have not stopped any CAM therapies since March 2014***
    ☐ I don't think it helped
    ☐ It was too expensive
    ☐ It is no longer available
    ☐ I experienced negative side effects
    ☐ I didn't like the therapy
    ☐ It was too difficult/required assistance
    ☐ I heard from a trusted source that it wasn't effective
    ☐ I read a guideline
    ☐ Other (please describe): _______________________________

Recently, the American Academy of Neurology (AAN) published an evidence-based clinical practice guideline about CAM treatments for MS.

A **clinical practice guideline** is a document that helps health care providers make decisions. Guidelines look at all the ways to diagnose and treat a disease, and this helps health care providers make a good decision for a patient. Guidelines from the AAN are developed by neurologists and other experts. The guidelines are based on a careful review of all available scientific studies for a specific brain disease.

21. Did you know that the AAN has developed a guideline about the use of CAM in MS?
    - ☐ Yes
    - ☐ No ⟶ **Please continue with Question 27 on Page 13.**

22. If you answered yes, can you tell us **where you heard** of the guideline? ***Please check all that apply.***
    - ☐ American Academy of Neurology (AAN) website
    - ☐ Email sent to me
    - ☐ Facebook
    - ☐ Friend or family member
    - ☐ Google+
    - ☐ Internet research
    - ☐ LinkedIn
    - ☐ My patient organization (which one?): ______________________________
    - ☐ My primary care provider
    - ☐ My neurologist
    - ☐ *Neurology Now*® article or website
    - ☐ News article (which news source?): ________________________________
    - ☐ Pinterest
    - ☐ Twitter
    - ☐ YouTube
    - ☐ Website (please identify): __________________________________
    - ☐ YouTube
    - ☐ Other (please describe): ____

23. Have you read the guideline or the summary of the guideline? ***Please check all that apply.***
    - ☐ Yes, I read the guideline ⟶ **Please continue with Question 24.**
    - ☐ Yes, I read the summary ⟶ **Please continue with Question 24.**
    - ☐ No, I did not read it, but I intend to ⟶ **Please continue with Question 26 on Page 12.**
    - ☐ No, I did not read the guideline or the summary ⟶ **Please continue with Question 27 on Page 13.**

24. If you read the guideline or the summary, how did you find the guideline?
Please check all that apply.
- ☐ American Academy of Neurology (AAN) website
- ☐ Email sent to me
- ☐ Facebook
- ☐ Google+
- ☐ I got it from a friend/family member
- ☐ I got it from my primary care provider
- ☐ I got it from my neurologist
- ☐ LinkedIn
- ☐ *Neurology Now* article or website
- ☐ Pinterest
- ☐ Twitter
- ☐ Website (please identify): ____
- ☐ YouTube
- ☐ Other (please describe): ____

25. Do you think the information in the guideline is:
- ☐ Very trustworthy
- ☐ Somewhat trustworthy
- ☐ Somewhat untrustworthy
- ☐ Not at all trustworthy
- ☐ Not sure

NOW GO TO QUESTION 27, PAGE 13.

26. If you answered "no, but I intend to," where will you go to find the guideline?
Please check all that apply.
- ☐ American Academy of Neurology (AAN) website
- ☐ Email sent to me
- ☐ Facebook
- ☐ Google+
- ☐ I will get it from a friend/family member
- ☐ I will get it from my primary care provider
- ☐ I will get it from my neurologist
- ☐ LinkedIn
- ☐ *Neurology Now* article or website
- ☐ Pinterest
- ☐ Twitter
- ☐ Website (please identify): ____
- ☐ YouTube
- ☐ Other (please describe): ____

The final set of questions is about you.

27. How long has it been since you were diagnosed with MS?
    - ☐ Less than 1 year
    - ☐ 1 – 5 years
    - ☐ 6 – 10 years
    - ☐ 11 – 20 years
    - ☐ More than 20 years

28. Are you able to walk?
    - ☐ I am walking without assistance
    - ☐ I am walking with assistance
    - ☐ I cannot walk; I rely on a wheelchair

29. Do you have problems with your vision?
    - ☐ I have no problems with my vision
    - ☐ I have some visual impairment
    - ☐ I have severe visual impairment/blindness

30. Do you have difficulty using your hands?
    - ☐ I have no difficulty using my hands
    - ☐ I have some difficulty using my hands
    - ☐ I have severe difficulty using my hands
    - ☐ I cannot use my hands

31. How old are you?
    ________ years old

32. What is your gender?
    - ☐ Female
    - ☐ Male
    - ☐ Prefer not to answer

33. Do you consider yourself Hispanic or Latino/a?
    - ☐ Yes, Hispanic or Latino/a
    - ☐ No

34. What is your race? ***Please check all that apply.***
    - ☐ American Indian or Native American
    - ☐ Asian
    - ☐ Black or African American
    - ☐ Hawaiian or other Pacific Islander
    - ☐ White
    - ☐ Other (please specify):

      ____

35. What is your highest level of education?
    - ☐ Did not attend high school
    - ☐ Some high school education
    - ☐ High school diploma
    - ☐ Some college education
    - ☐ College diploma
    - ☐ Some postgraduate education
    - ☐ Postgraduate diploma

Thank you!
Please return your survey in the self-addressed return envelope.

Patient Post-Dissemination Survey 2

AMERICAN ACADEMY OF NEUROLOGY INSTITUTE

Use of Complementary and Alternative Treatments in Multiple Sclerosis

This survey asks about your attitudes regarding complementary and alternative medicine (CAM) treatments for multiple sclerosis (MS), how you get your information about MS, and how you think your MS should be treated. This survey should take no more than 5–10 minutes to complete. We appreciate your time and feedback.

Unless otherwise instructed, please select one (1) response that best reflects your answer.
There are no right or wrong answers.

If you are a caregiver, please fill out the survey *as if you are the patient.*
☐ **Check here if this survey was completed by a patient representative or caregiver.**

For questions, please contact:
Pushpa Narayanaswami, MBBS, DM, FAAN
Neurology TCC-8, BIDMC
330 Brookline Avenue
Boston, MA 02215
Phone: (617) 667-8130
Fax: (617) 667-3175
Email: pnarayan@bidmc.harvard.edu

Before we begin, we have a few questions about your use of the Internet.

1. How often are you on the Internet?
   - ☐ I rarely use a computer or the Internet
   - ☐ I use the Internet once a week
   - ☐ I use the Internet several times per week
   - ☐ I use the Internet every day

2. Do you have ***any*** disability that makes it hard for you to use the Internet?
   - ☐ Yes
   - ☐ No

3. If you use the Internet, what is your favorite site to go to learn about MS? ______________________

4. Please name other sites you visit for MS information:

   ____

   ____

   ____

   ____

   ____

   ____

The National Center for Complementary and Alternative Medicine (NCCAM) defines complementary and alternative medicine (CAM) as medical practices and products that are not generally considered part of conventional medicine.

- ***Conventional medicine*** is medicine practiced by medical doctors and health professionals such as physical therapists, psychologists, and registered nurses. Examples of conventional medicine are vaccines, prescriptions, and surgery.
- ***Complementary medicine*** is non-conventional medicine practiced **together with** conventional medicine. An example is using acupuncture **in addition to** medication to help lessen pain.
- ***Alternative medicine*** refers to use of non-conventional medicine practiced **instead of** conventional medicine. An example of "non-conventional" therapy is using an herbal supplement such as ginkgo biloba for memory.

5. Have you ***heard of*** CAM therapies before?
   - ☐ Yes
   - ☐ No

Below is a list of CAM therapies. For each therapy listed below, please tell us whether you have ***ever used*** the therapy and whether the therapy ***helped to ease*** your symptoms.

6. For each therapy below, please answer each question.

	Have you ***ever used*** this therapy?		***If you used it***, did this therapy help ease your symptoms?	
	Yes	No	Yes	No
Acupuncture	☐	☐	☐	☐
Amalgam replacement	☐	☐	☐	☐
Bee venom (sting)	☐	☐	☐	☐
Biofeedback	☐	☐	☐	☐
Cannabis extract (e.g., Cesamet, Marinol)	☐	☐	☐	☐
Carnitine	☐	☐	☐	☐
Chelation	☐	☐	☐	☐
Chiropractic care	☐	☐	☐	☐
Creatine	☐	☐	☐	☐
Ginkgo biloba	☐	☐	☐	☐
Glucosamine	☐	☐	☐	☐
Hippotherapy (therapeutic horseback riding)	☐	☐	☐	☐
Hyperbaric oxygen	☐	☐	☐	☐
Inosine	☐	☐	☐	☐
Linoleic acid	☐	☐	☐	☐
Lofepramine	☐	☐	☐	☐
Low-dose naltrexone	☐	☐	☐	☐
Magnetic therapy	☐	☐	☐	☐
Massage therapy	☐	☐	☐	☐
Mindfulness	☐	☐	☐	☐
Music therapy	☐	☐	☐	☐
Naturopathic medicine	☐	☐	☐	☐
Neural therapy	☐	☐	☐	☐
Omega-3 fatty acid supplementation (e.g., fish oil)	☐	☐	☐	☐

Please continue to tell us whether ***you have ever used*** these CAM therapies and ***if you used it***, whether it ***helped to ease*** your symptoms.

	Have you ***ever used*** this therapy?		***If you used it***, did this therapy help ease your symptoms?	
	Yes	No	Yes	No
Padma 28	☐	☐	☐	☐
Phenylalanine	☐	☐	☐	☐
Progressive muscle relaxation	☐	☐	☐	☐
Reflexology	☐	☐	☐	☐
Smoking marijuana	☐	☐	☐	☐
Tai chi	☐	☐	☐	☐
Threonine	☐	☐	☐	☐
Transdermal histamine (e.g., Prokarin patch)	☐	☐	☐	☐
Yoga	☐	☐	☐	☐
Other (please describe): ____________________ ____________________ ____________________	☐	☐	☐	☐

The next set of questions is about specific CAM therapies. **Please select one (1) response that best reflects the extent to which you agree or disagree with each of the statements below.**

	**Agree**	**Somewhat agree**	**Somewhat disagree**	**Disagree**	**Not sure**
7. Magnetic therapy is effective for treating fatigue in people with MS.	☐	☐	☐	☐	☐
8. Taking oral cannabis extract is effective for treating tremors in people with MS.	☐	☐	☐	☐	☐
9. Smoking marijuana (cannabis) is effective for treating balance, posture, or pain in people with MS.	☐	☐	☐	☐	☐
10. Taking ginkgo biloba orally is effective for improving memory in people with MS.	☐	☐	☐	☐	☐
11. Hyperbaric oxygen is effective for improving symptoms in people with MS.	☐	☐	☐	☐	☐
12. Bee sting therapy is effective for reducing MRI lesions in people with MS.	☐	☐	☐	☐	☐

The next set of questions asks about your ***intentions*** to use CAM therapies.

13. Do you ***plan to initiate discussions*** *about* any of these CAM therapies for MS with your doctor who treats your MS? ***Please check all that apply*** *or select "No" if you do not plan to initiate any discussions about any CAM therapies with your doctor.*

    ☐ ***No, I do not plan to initiate any discussions about any CAM therapies with my doctor who treats my MS.***

☐ Acupuncture
☐ Amalgam replacement
☐ Bee venom (sting)
☐ Biofeedback
☐ Cannabis extract (e.g., Cesamet, Marinol)
☐ Carnitine
☐ Chelation
☐ Chiropractic care
☐ Creatine
☐ Ginkgo biloba
☐ Glucosamine
☐ Hippotherapy (therapeutic horseback riding)
☐ Hyperbaric oxygen
☐ Inosine
☐ Linoleic acid
☐ Lofepramine
☐ Low-dose naltrexone
☐ Magnetic therapy
☐ Massage therapy
☐ Mindfulness
☐ Music therapy
☐ Naturopathic medicine
☐ Neural therapy
☐ Omega-3 fatty acid supplementation (e.g., fish oil)
☐ Padma 28
☐ Phenylalanine
☐ Progressive muscle relaxation
☐ Reflexology
☐ Smoking marijuana
☐ Tai chi
☐ Threonine
☐ Transdermal histamine (e.g., Prokarin patch)
☐ Yoga
☐ Other (please describe):
____
____
____

14. Do you ***intend to start using*** any CAM therapies for treatment of your MS?
    ☐ Yes
    ☐ No

15. Do you ***intend to stop using*** any CAM therapies for treatment of your MS?
    ☐ Yes
    ☐ No

If you said "No" to BOTH 14 and 15, GO TO QUESTION 17, PAGE 8.

16. Which CAM therapies do you ***intend to start using*** or ***stop using*** for treatment of your MS?

	I intend to ***start using*** this CAM therapy.		I intend to ***stop using*** this CAM therapy.	
	Yes	No	Yes	No
Acupuncture	☐	☐	☐	☐
Amalgam replacement	☐	☐	☐	☐
Bee venom (sting)	☐	☐	☐	☐
Biofeedback	☐	☐	☐	☐
Cannabis extract (e.g., Cesamet, Marinol)	☐	☐	☐	☐
Carnitine	☐	☐	☐	☐
Chelation	☐	☐	☐	☐
Chiropractic care	☐	☐	☐	☐
Creatine	☐	☐	☐	☐
Ginkgo biloba	☐	☐	☐	☐
Glucosamine	☐	☐	☐	☐
Hippotherapy (therapeutic horseback riding)	☐	☐	☐	☐
Hyperbaric oxygen	☐	☐	☐	☐
Inosine	☐	☐	☐	☐
Linoleic acid	☐	☐	☐	☐
Lofepramine	☐	☐	☐	☐
Low-dose naltrexone	☐	☐	☐	☐
Magnetic therapy	☐	☐	☐	☐
Massage therapy	☐	☐	☐	☐
Mindfulness	☐	☐	☐	☐
Music therapy	☐	☐	☐	☐
Naturopathic medicine	☐	☐	☐	☐
Neural therapy	☐	☐	☐	☐
Omega-3 fatty acid supplementation (e.g., fish oil)	☐	☐	☐	☐
Padma 28	☐	☐	☐	☐
Phenylalanine	☐	☐	☐	☐
Progressive muscle relaxation	☐	☐	☐	☐
Reflexology	☐	☐	☐	☐
Smoking marijuana	☐	☐	☐	☐
Tai chi	☐	☐	☐	☐
Threonine	☐	☐	☐	☐
Transdermal histamine (e.g., Prokarin patch)	☐	☐	☐	☐
Yoga	☐	☐	☐	☐
Other (please describe): ____________________________ ____________________________ ____________________________	☐	☐	☐	☐

The next set of questions is about your ***actual behavior*** in relation to CAM therapies.

17. Since *late June 2014*, have you ***discussed*** any of these CAM therapies for MS with your doctor who treats your MS? ***Please check all that apply*** *or select "No" if you have not discussed any CAM therapies with your doctor.*

    ◻ ***No, I have not discussed any CAM therapies with my doctor who treats my MS.***

☐ Acupuncture
☐ Amalgam replacement
☐ Bee venom (sting)
☐ Biofeedback
☐ Cannabis extract (e.g., Cesamet, Marinol)
☐ Carnitine
☐ Chelation
☐ Chiropractic care
☐ Creatine
☐ Ginkgo biloba
☐ Glucosamine
☐ Hippotherapy (therapeutic horseback riding)
☐ Hyperbaric oxygen
☐ Inosine
☐ Linoleic acid
☐ Lofepramine
☐ Low-dose naltrexone
☐ Magnetic therapy
☐ Massage therapy
☐ Mindfulness
☐ Music therapy
☐ Naturopathic medicine
☐ Neural therapy
☐ Omega-3 fatty acid supplementation (e.g., fish oil)
☐ Padma 28
☐ Phenylalanine
☐ Progressive muscle relaxation
☐ Reflexology
☐ Smoking marijuana
☐ Tai chi
☐ Threonine
☐ Transdermal histamine (e.g., Prokarin patch)
☐ Yoga
☐ Other (please describe):
____
____
____

18. Since *late June 2014*, have you ***started using*** or ***stopped using*** any of these CAM therapies for MS?

	I have ***started using*** this CAM therapy since *late June 2014*		I have ***stopped using*** this CAM therapy since *late June 2014*	
	Yes	No	Yes	No
Acupuncture	☐	☐	☐	☐
Amalgam replacement	☐	☐	☐	☐
Bee venom (sting)	☐	☐	☐	☐
Biofeedback	☐	☐	☐	☐
Cannabis extract (e.g., Cesamet, Marinol)	☐	☐	☐	☐
Carnitine	☐	☐	☐	☐
Chelation	☐	☐	☐	☐
Chiropractic care	☐	☐	☐	☐
Creatine	☐	☐	☐	☐
Ginkgo biloba	☐	☐	☐	☐
Glucosamine	☐	☐	☐	☐
Hippotherapy (therapeutic horseback riding)	☐	☐	☐	☐
Hyperbaric oxygen	☐	☐	☐	☐
Inosine	☐	☐	☐	☐
Linoleic acid	☐	☐	☐	☐
Lofepramine	☐	☐	☐	☐
Low-dose naltrexone	☐	☐	☐	☐
Magnetic therapy	☐	☐	☐	☐
Massage therapy	☐	☐	☐	☐
Mindfulness	☐	☐	☐	☐
Music therapy	☐	☐	☐	☐
Naturopathic medicine	☐	☐	☐	☐
Neural therapy	☐	☐	☐	☐
Omega-3 fatty acid supplementation (e.g., fish oil)	☐	☐	☐	☐
Padma 28	☐	☐	☐	☐
Phenylalanine	☐	☐	☐	☐
Progressive muscle relaxation	☐	☐	☐	☐
Reflexology	☐	☐	☐	☐
Smoking marijuana	☐	☐	☐	☐
Tai chi	☐	☐	☐	☐
Threonine	☐	☐	☐	☐
Transdermal histamine (e.g., Prokarin patch)	☐	☐	☐	☐
Yoga	☐	☐	☐	☐
Other (please describe): ____________________________ ____________________________ ____________________________	☐	☐	☐	☐

We know that there are many different reasons why people ***start or stop using*** CAM therapies. We would like to know why you ***started*** or ***stopped*** using the therapy (or therapies) you selected previously.

If you started or stopped using more than one (1) CAM therapy, please ***check all the reasons that apply***. Even though you may have different reasons for *starting or stopping* the use of different CAM therapies, we only need to know what your reasons are overall.

19. For all the therapies you ***started*** since *late June 2014*:

    What were your overall reasons for choosing ***to start*** this therapy (or therapies)?
    Please check all that apply.
    ☐ ***I have not started any CAM therapies since late June 2014***
    ☐ I hoped it would improve/help manage my MS symptoms
    ☐ It was recommended by a friend or family member
    ☐ It was recommended by a physician/care provider
    ☐ I learned about it from TV
    ☐ I learned about it from the Internet
    ☐ I read a clinical practice guideline
    ☐ My insurance now pays for it
    ☐ It has recently become available in my area
    ☐ It has recently become legal in my area
    ☐ Other (please describe):________________________

20. For all the therapies you ***stopped*** since *late June 2014*:

    What were your overall reasons for choosing ***to stop*** this therapy (or therapies)?
    Please check all that apply.
    ☐ ***I have not stopped any CAM therapies since late June 2014***
    ☐ I don't think it helped
    ☐ It was too expensive
    ☐ It is no longer available
    ☐ I experienced negative side effects
    ☐ I didn't like the therapy
    ☐ It was too difficult/required assistance
    ☐ I heard from a trusted source that it wasn't effective
    ☐ I read a guideline
    ☐ Other (please describe): _______________________________

Recently, the American Academy of Neurology (AAN) published an evidence-based clinical practice guideline about CAM treatments for MS.

A **clinical practice guideline** is a document that helps health care providers make decisions. Guidelines look at all the ways to diagnose and treat a disease, and this helps health care providers make a good decision for a patient. Guidelines from the AAN are developed by neurologists and other experts. The guidelines are based on a careful review of all available scientific studies for a specific brain disease.

21. Did you know that the AAN has developed a guideline about the use of CAM in MS?
   - ☐ Yes
   - ☐ No ⟶ **Please continue with Question 27 on Page 13.**

22. If you answered yes, can you tell us **where you heard** of the guideline? ***Please check all that apply.***
   - ☐ American Academy of Neurology (AAN) website
   - ☐ Email sent to me
   - ☐ Facebook
   - ☐ Friend or family member
   - ☐ Google+
   - ☐ Internet research
   - ☐ LinkedIn
   - ☐ My patient organization (which one?): ______________________________
   - ☐ My primary care provider
   - ☐ My neurologist
   - ☐ *Neurology Now®* article or website
   - ☐ News article (which news source?): ________________________________
   - ☐ Pinterest
   - ☐ Twitter
   - ☐ YouTube
   - ☐ Website (please identify): __________________________________
   - ☐ YouTube
   - ☐ Other (please describe): ____

23. Have you read the guideline or the summary of the guideline? ***Please check all that apply.***
   - ☐ Yes, I read the guideline ⟶ **Please continue with Question 24.**
   - ☐ Yes, I read the summary ⟶ **Please continue with Question 24.**
   - ☐ No, I did not read it, but I intend to ⟶ **Please continue with Question 26 on Page 12.**
   - ☐ No, I did not read the guideline or the summary ⟶ **Please continue with Question 27 on Page 13.**

24. If you read the guideline or the summary, how did you find the guideline?
Please check all that apply.
- ☐ American Academy of Neurology (AAN) website
- ☐ Email sent to me
- ☐ Facebook
- ☐ Google+
- ☐ I got it from a friend/family member
- ☐ I got it from my primary care provider
- ☐ I got it from my neurologist
- ☐ LinkedIn
- ☐ *Neurology Now* article or website
- ☐ Pinterest
- ☐ Twitter
- ☐ Website (please identify): ______________________________________
- ☐ YouTube
- ☐ Other (please describe): ____

25. Do you think the information in the guideline is:
- ☐ Very trustworthy
- ☐ Somewhat trustworthy
- ☐ Somewhat untrustworthy
- ☐ Not at all trustworthy
- ☐ Not sure

NOW GO TO QUESTION 27, PAGE 13.

26. If you answered "no, but I intend to," where will you go to find the guideline?
Please check all that apply.
- ☐ American Academy of Neurology (AAN) website
- ☐ Email sent to me
- ☐ Facebook
- ☐ Google+
- ☐ I will get it from a friend/family member
- ☐ I will get it from my primary care provider
- ☐ I will get it from my neurologist
- ☐ LinkedIn
- ☐ *Neurology Now* article or website
- ☐ Pinterest
- ☐ Twitter
- ☐ Website (please identify): ____
- ☐ YouTube
- ☐ Other (please describe): ____

The final set of questions is about you.

27. How long has it been since you were diagnosed with MS?
    - ☐ Less than 1 year
    - ☐ 1 – 5 years
    - ☐ 6 – 10 years
    - ☐ 11 – 20 years
    - ☐ More than 20 years

28. Are you able to walk?
    - ☐ I am walking without assistance
    - ☐ I am walking with assistance
    - ☐ I cannot walk; I rely on a wheelchair

29. Do you have problems with your vision?
    - ☐ I have no problems with my vision
    - ☐ I have some visual impairment
    - ☐ I have severe visual impairment/blindness

30. Do you have difficulty using your hands?
    - ☐ I have no difficulty using my hands
    - ☐ I have some difficulty using my hands
    - ☐ I have severe difficulty using my hands
    - ☐ I cannot use my hands

31. How old are you?
    ________ years old

32. What is your gender?
    - ☐ Female
    - ☐ Male
    - ☐ Prefer not to answer

33. Do you consider yourself Hispanic or Latino/a?
    - ☐ Yes, Hispanic or Latino/a
    - ☐ No

34. What is your race? ***Please check all that apply.***
    - ☐ American Indian or Native American
    - ☐ Asian
    - ☐ Black or African American
    - ☐ Hawaiian or other Pacific Islander
    - ☐ White
    - ☐ Other (please specify):

      ____

35. What is your highest level of education?
    - ☐ Did not attend high school
    - ☐ Some high school education
    - ☐ High school diploma
    - ☐ Some college education
    - ☐ College diploma
    - ☐ Some postgraduate education
    - ☐ Postgraduate diploma

Thank you!
Please return your survey in the self-addressed return envelope.

Patient Post-Dissemination Survey 3

Use of Complementary and Alternative Treatments in Multiple Sclerosis

This survey asks about your attitudes regarding complementary and alternative medicine (CAM) treatments for multiple sclerosis (MS), how you get your information about MS, and how you think your MS should be treated. This survey should take no more than 5–10 minutes to complete. We appreciate your time and feedback.

Unless otherwise instructed, please select one (1) response that best reflects your answer. **There are no right or wrong answers.**

If you are a caregiver, please fill out the survey *as if you are the patient.*
☐ **Check here if this survey was completed by a patient representative or caregiver.**

For questions, please contact:
Pushpa Narayanaswami, MBBS, DM, FAAN
Neurology TCC-8, BIDMC
330 Brookline Avenue
Boston, MA 02215
Phone: (617) 667-8130
Fax: (617) 667-3175
Email: pnarayan@bidmc.harvard.edu

Before we begin, we have a few questions about your use of the Internet.

1. How often are you on the Internet?
   - ☐ I rarely use a computer or the Internet
   - ☐ I use the Internet once a week
   - ☐ I use the Internet several times per week
   - ☐ I use the Internet every day

2. Do you have ***any*** disability that makes it hard for you to use the Internet?
   - ☐ Yes
   - ☐ No

3. If you use the Internet, what is your favorite site to go to learn about MS? ______________________

4. Please name other sites you visit for MS information:

   ____

   ____

   ____

   ____

   ____

   ____

The National Center for Complementary and Alternative Medicine (NCCAM) defines complementary and alternative medicine (CAM) as medical practices and products that are not generally considered part of conventional medicine.

- ***Conventional medicine*** is medicine practiced by medical doctors and health professionals such as physical therapists, psychologists, and registered nurses. Examples of conventional medicine are vaccines, prescriptions, and surgery.
- ***Complementary medicine*** is non-conventional medicine practiced **together with** conventional medicine. An example is using acupuncture **in addition to** medication to help lessen pain.
- ***Alternative medicine*** refers to use of non-conventional medicine practiced **instead of** conventional medicine. An example of "non-conventional" therapy is using an herbal supplement such as ginkgo biloba for memory.

5. Have you ***heard of*** CAM therapies before?
   - ☐ Yes
   - ☐ No

Below is a list of CAM therapies. For each therapy listed below, please tell us whether you have ***ever used*** the therapy and whether the therapy ***helped to ease*** your symptoms.

6. For each therapy below, please answer each question.

	Have you ***ever used*** this therapy?		***If you used it***, did this therapy help ease your symptoms?	
	Yes	No	Yes	No
Acupuncture	☐	☐	☐	☐
Amalgam replacement	☐	☐	☐	☐
Bee venom (sting)	☐	☐	☐	☐
Biofeedback	☐	☐	☐	☐
Cannabis extract (e.g., Cesamet, Marinol)	☐	☐	☐	☐
Carnitine	☐	☐	☐	☐
Chelation	☐	☐	☐	☐
Chiropractic care	☐	☐	☐	☐
Creatine	☐	☐	☐	☐
Ginkgo biloba	☐	☐	☐	☐
Glucosamine	☐	☐	☐	☐
Hippotherapy (therapeutic horseback riding)	☐	☐	☐	☐
Hyperbaric oxygen	☐	☐	☐	☐
Inosine	☐	☐	☐	☐
Linoleic acid	☐	☐	☐	☐
Lofepramine	☐	☐	☐	☐
Low-dose naltrexone	☐	☐	☐	☐
Magnetic therapy	☐	☐	☐	☐
Massage therapy	☐	☐	☐	☐
Mindfulness	☐	☐	☐	☐
Music therapy	☐	☐	☐	☐
Naturopathic medicine	☐	☐	☐	☐
Neural therapy	☐	☐	☐	☐
Omega-3 fatty acid supplementation (e.g., fish oil)	☐	☐	☐	☐

Please continue to tell us whether ***you have ever used*** these CAM therapies and ***if you used it***, whether it ***helped to ease*** your symptoms.

	Have you ***ever used*** this therapy?		***If you used it***, did this therapy help ease your symptoms?	
	Yes	No	Yes	No
Padma 28	☐	☐	☐	☐
Phenylalanine	☐	☐	☐	☐
Progressive muscle relaxation	☐	☐	☐	☐
Reflexology	☐	☐	☐	☐
Smoking marijuana	☐	☐	☐	☐
Tai chi	☐	☐	☐	☐
Threonine	☐	☐	☐	☐
Transdermal histamine (e.g., Prokarin patch)	☐	☐	☐	☐
Yoga	☐	☐	☐	☐
Other (please describe): ____________________ ____________________ ____________________	☐	☐	☐	☐

The next set of questions is about specific CAM therapies. **Please select one (1) response that best reflects the extent to which you agree or disagree with each of the statements below.**

	**Agree**	**Somewhat agree**	**Somewhat disagree**	**Disagree**	**Not sure**
7. Magnetic therapy is effective for treating fatigue in people with MS.	☐	☐	☐	☐	☐
8. Taking oral cannabis extract is effective for treating tremors in people with MS.	☐	☐	☐	☐	☐
9. Smoking marijuana (cannabis) is effective for treating balance, posture, or pain in people with MS.	☐	☐	☐	☐	☐
10. Taking ginkgo biloba orally is effective for improving memory in people with MS.	☐	☐	☐	☐	☐
11. Hyperbaric oxygen is effective for improving symptoms in people with MS.	☐	☐	☐	☐	☐
12. Bee sting therapy is effective for reducing MRI lesions in people with MS.	☐	☐	☐	☐	☐

The next set of questions asks about your ***intentions*** to use CAM therapies.

13. Do you ***plan to initiate discussions*** *about* any of these CAM therapies for MS with your doctor who treats your MS? ***Please check all that apply*** *or select "No" if you do not plan to initiate any discussions about any CAM therapies with your doctor.*

- □ ***No, I do not plan to initiate any discussions about any CAM therapies with my doctor who treats my MS.***

- ☐ Acupuncture
- ☐ Amalgam replacement
- ☐ Bee venom (sting)
- ☐ Biofeedback
- ☐ Cannabis extract (e.g., Cesamet, Marinol)
- ☐ Carnitine
- ☐ Chelation
- ☐ Chiropractic care
- ☐ Creatine
- ☐ Ginkgo biloba
- ☐ Glucosamine
- ☐ Hippotherapy (therapeutic horseback riding)
- ☐ Hyperbaric oxygen
- ☐ Inosine
- ☐ Linoleic acid
- ☐ Lofepramine
- ☐ Low-dose naltrexone
- ☐ Magnetic therapy
- ☐ Massage therapy
- ☐ Mindfulness
- ☐ Music therapy
- ☐ Naturopathic medicine
- ☐ Neural therapy
- ☐ Omega-3 fatty acid supplementation (e.g., fish oil)
- ☐ Padma 28
- ☐ Phenylalanine
- ☐ Progressive muscle relaxation
- ☐ Reflexology
- ☐ Smoking marijuana
- ☐ Tai chi
- ☐ Threonine
- ☐ Transdermal histamine (e.g., Prokarin patch)
- ☐ Yoga
- ☐ Other (please describe): ____________________________________
  ____
  ____

14. Do you ***intend to start using*** any CAM therapies for treatment of your MS?
- ☐ Yes
- ☐ No

15. Do you ***intend to stop using*** any CAM therapies for treatment of your MS?
- ☐ Yes
- ☐ No

If you said "No" to BOTH 14 and 15, GO TO QUESTION 17, PAGE 8.

16. Which CAM therapies do you ***intend to start using*** or ***stop using*** for treatment of your MS?

	I intend to ***start using*** this CAM therapy.		I intend to ***stop using*** this CAM therapy.	
	Yes	No	Yes	No
Acupuncture	☐	☐	☐	☐
Amalgam replacement	☐	☐	☐	☐
Bee venom (sting)	☐	☐	☐	☐
Biofeedback	☐	☐	☐	☐
Cannabis extract (e.g., Cesamet, Marinol)	☐	☐	☐	☐
Carnitine	☐	☐	☐	☐
Chelation	☐	☐	☐	☐
Chiropractic care	☐	☐	☐	☐
Creatine	☐	☐	☐	☐
Ginkgo biloba	☐	☐	☐	☐
Glucosamine	☐	☐	☐	☐
Hippotherapy (therapeutic horseback riding)	☐	☐	☐	☐
Hyperbaric oxygen	☐	☐	☐	☐
Inosine	☐	☐	☐	☐
Linoleic acid	☐	☐	☐	☐
Lofepramine	☐	☐	☐	☐
Low-dose naltrexone	☐	☐	☐	☐
Magnetic therapy	☐	☐	☐	☐
Massage therapy	☐	☐	☐	☐
Mindfulness	☐	☐	☐	☐
Music therapy	☐	☐	☐	☐
Naturopathic medicine	☐	☐	☐	☐
Neural therapy	☐	☐	☐	☐
Omega-3 fatty acid supplementation (e.g., fish oil)	☐	☐	☐	☐
Padma 28	☐	☐	☐	☐
Phenylalanine	☐	☐	☐	☐
Progressive muscle relaxation	☐	☐	☐	☐
Reflexology	☐	☐	☐	☐
Smoking marijuana	☐	☐	☐	☐
Tai chi	☐	☐	☐	☐
Threonine	☐	☐	☐	☐
Transdermal histamine (e.g., Prokarin patch)	☐	☐	☐	☐
Yoga	☐	☐	☐	☐
Other (please describe): _________________________ _________________________ _________________________	☐	☐	☐	☐

The next set of questions is about your ***actual behavior*** in relation to CAM therapies.

17. Since *March 2014*, have you **discussed** any of these CAM therapies for MS with your doctor who treats your MS? ***Please check all that apply*** *or select "No" if you have not discussed any CAM therapies with your doctor.*

    - ☐ ***No, I have not discussed any CAM therapies with my doctor who treats my MS.***

- ☐ Acupuncture
- ☐ Amalgam replacement
- ☐ Bee venom (sting)
- ☐ Biofeedback
- ☐ Cannabis extract (e.g., Cesamet, Marinol)
- ☐ Carnitine
- ☐ Chelation
- ☐ Chiropractic care
- ☐ Creatine
- ☐ Ginkgo biloba
- ☐ Glucosamine
- ☐ Hippotherapy (therapeutic horseback riding)
- ☐ Hyperbaric oxygen
- ☐ Inosine
- ☐ Linoleic acid
- ☐ Lofepramine
- ☐ Low-dose naltrexone
- ☐ Magnetic therapy
- ☐ Massage therapy
- ☐ Mindfulness
- ☐ Music therapy
- ☐ Naturopathic medicine
- ☐ Neural therapy
- ☐ Omega-3 fatty acid supplementation (e.g., fish oil)
- ☐ Padma 28
- ☐ Phenylalanine
- ☐ Progressive muscle relaxation
- ☐ Reflexology
- ☐ Smoking marijuana
- ☐ Tai chi
- ☐ Threonine
- ☐ Transdermal histamine (e.g., Prokarin patch)
- ☐ Yoga
- ☐ Other (please describe): ____________________________________
  ____
  ____

18. Since *March 2014*, have you ***started using*** or ***stopped using*** any of these CAM therapies for MS?

	I have ***started using*** this CAM therapy since *March 2014*		I have ***stopped using*** this CAM therapy since *March 2014*	
	Yes	No	Yes	No
Acupuncture	☐	☐	☐	☐
Amalgam replacement	☐	☐	☐	☐
Bee venom (sting)	☐	☐	☐	☐
Biofeedback	☐	☐	☐	☐
Cannabis extract (e.g., Cesamet, Marinol)	☐	☐	☐	☐
Carnitine	☐	☐	☐	☐
Chelation	☐	☐	☐	☐
Chiropractic care	☐	☐	☐	☐
Creatine	☐	☐	☐	☐
Ginkgo biloba	☐	☐	☐	☐
Glucosamine	☐	☐	☐	☐
Hippotherapy (therapeutic horseback riding)	☐	☐	☐	☐
Hyperbaric oxygen	☐	☐	☐	☐
Inosine	☐	☐	☐	☐
Linoleic acid	☐	☐	☐	☐
Lofepramine	☐	☐	☐	☐
Low-dose naltrexone	☐	☐	☐	☐
Magnetic therapy	☐	☐	☐	☐
Massage therapy	☐	☐	☐	☐
Mindfulness	☐	☐	☐	☐
Music therapy	☐	☐	☐	☐
Naturopathic medicine	☐	☐	☐	☐
Neural therapy	☐	☐	☐	☐
Omega-3 fatty acid supplementation (e.g., fish oil)	☐	☐	☐	☐
Padma 28	☐	☐	☐	☐
Phenylalanine	☐	☐	☐	☐
Progressive muscle relaxation	☐	☐	☐	☐
Reflexology	☐	☐	☐	☐
Smoking marijuana	☐	☐	☐	☐
Tai chi	☐	☐	☐	☐
Threonine	☐	☐	☐	☐
Transdermal histamine (e.g., Prokarin patch)	☐	☐	☐	☐
Yoga	☐	☐	☐	☐
Other (please describe): ____________________________ ____________________________ ____________________________	☐	☐	☐	☐

We know that there are many different reasons why people ***start or stop using*** CAM therapies. We would like to know why you ***started*** or ***stopped*** using the therapy (or therapies) you selected previously.

If you started or stopped using more than one (1) CAM therapy, please ***check all the reasons that apply***. Even though you may have different reasons for *starting or stopping* the use of different CAM therapies, we only need to know what your reasons are overall.

19. For all the therapies you ***started*** since *March 2014*:

    What were your overall reasons for choosing ***to start*** this therapy (or therapies)?
    Please check all that apply.
    ☐ ***I have not started any CAM therapies since March 2014***
    ☐ I hoped it would improve/help manage my MS symptoms
    ☐ It was recommended by a friend or family member
    ☐ It was recommended by a physician/care provider
    ☐ I learned about it from TV
    ☐ I learned about it from the Internet
    ☐ I read a clinical practice guideline
    ☐ My insurance now pays for it
    ☐ It has recently become available in my area
    ☐ It has recently become legal in my area
    ☐ Other (please describe):________________________

20. For all the therapies you ***stopped*** since *March 2014*:

    What were your overall reasons for choosing ***to stop*** this therapy (or therapies)?
    Please check all that apply.
    ☐ ***I have not stopped any CAM therapies since March 2014***
    ☐ I don’t think it helped
    ☐ It was too expensive
    ☐ It is no longer available
    ☐ I experienced negative side effects
    ☐ I didn’t like the therapy
    ☐ It was too difficult/required assistance
    ☐ I heard from a trusted source that it wasn’t effective
    ☐ I read a guideline
    ☐ Other (please describe): _______________________________

Recently, the American Academy of Neurology (AAN) published an evidence-based clinical practice guideline about CAM treatments for MS.

A **clinical practice guideline** is a document that helps health care providers make decisions. Guidelines look at all the ways to diagnose and treat a disease, and this helps health care providers make a good decision for a patient. Guidelines from the AAN are developed by neurologists and other experts. The guidelines are based on a careful review of all available scientific studies for a specific brain disease.

21. Did you know that the AAN has developed a guideline about the use of CAM in MS?
   - ☐ Yes
   - ☐ No ⟶ **Please continue with Question 27 on Page 13.**

22. If you answered yes, can you tell us **where you heard** of the guideline? ***Please check all that apply.***
   - ☐ American Academy of Neurology (AAN) website
   - ☐ Email sent to me
   - ☐ Facebook
   - ☐ Friend or family member
   - ☐ Google+
   - ☐ Internet research
   - ☐ LinkedIn
   - ☐ My patient organization (which one?): ______________________________
   - ☐ My primary care provider
   - ☐ My neurologist
   - ☐ *Neurology Now*® article or website
   - ☐ News article (which news source?): ________________________________
   - ☐ Pinterest
   - ☐ Twitter
   - ☐ YouTube
   - ☐ Website (please identify): __________________________________
   - ☐ YouTube
   - ☐ Other (please describe): ____

23. Have you read the guideline or the summary of the guideline? ***Please check all that apply.***
   - ☐ Yes, I read the guideline ⟶ **Please continue with Question 24.**
   - ☐ Yes, I read the summary ⟶ **Please continue with Question 24.**
   - ☐ No, I did not read it, but I intend to ⟶ **Please continue with Question 26 on Page 12.**
   - ☐ No, I did not read the guideline or the summary ⟶ **Please continue with Question 27 on Page 13.**

24. If you read the guideline or the summary, how did you find the guideline?
Please check all that apply.
- ☐ American Academy of Neurology (AAN) website
- ☐ Email sent to me
- ☐ Facebook
- ☐ Google+
- ☐ I got it from a friend/family member
- ☐ I got it from my primary care provider
- ☐ I got it from my neurologist
- ☐ LinkedIn
- ☐ *Neurology Now* article or website
- ☐ Pinterest
- ☐ Twitter
- ☐ Website (please identify): _______________________________________
- ☐ YouTube
- ☐ Other (please describe): ____

25. Do you think the information in the guideline is:
- ☐ Very trustworthy
- ☐ Somewhat trustworthy
- ☐ Somewhat untrustworthy
- ☐ Not at all trustworthy
- ☐ Not sure

NOW GO TO QUESTION 27, PAGE 13.

26. If you answered “no, but I intend to,” where will you go to find the guideline?
Please check all that apply.
- ☐ American Academy of Neurology (AAN) website
- ☐ Email sent to me
- ☐ Facebook
- ☐ Google+
- ☐ I will get it from a friend/family member
- ☐ I will get it from my primary care provider
- ☐ I will get it from my neurologist
- ☐ LinkedIn
- ☐ *Neurology Now* article or website
- ☐ Pinterest
- ☐ Twitter
- ☐ Website (please identify): ____
- ☐ YouTube
- ☐ Other (please describe): ____

The final set of questions is about you.

27. How long has it been since you were diagnosed with MS?
    - ☐ Less than 1 year
    - ☐ 1 – 5 years
    - ☐ 6 – 10 years
    - ☐ 11 – 20 years
    - ☐ More than 20 years

28. Are you able to walk?
    - ☐ I am walking without assistance
    - ☐ I am walking with assistance
    - ☐ I cannot walk; I rely on a wheelchair

29. Do you have problems with your vision?
    - ☐ I have no problems with my vision
    - ☐ I have some visual impairment
    - ☐ I have severe visual impairment/blindness

30. Do you have difficulty using your hands?
    - ☐ I have no difficulty using my hands
    - ☐ I have some difficulty using my hands
    - ☐ I have severe difficulty using my hands
    - ☐ I cannot use my hands

31. How old are you?
    ________ years old

32. What is your gender?
    - ☐ Female
    - ☐ Male
    - ☐ Prefer not to answer

33. Do you consider yourself Hispanic or Latino/a?
    - ☐ Yes, Hispanic or Latino/a
    - ☐ No

34. What is your race? ***Please check all that apply.***
    - ☐ American Indian or Native American
    - ☐ Asian
    - ☐ Black or African American
    - ☐ Hawaiian or other Pacific Islander
    - ☐ White
    - ☐ Other (please specify):

    ____

35. What is your highest level of education?
    - ☐ Did not attend high school
    - ☐ Some high school education
    - ☐ High school diploma
    - ☐ Some college education
    - ☐ College diploma
    - ☐ Some postgraduate education
    - ☐ Postgraduate diploma

Thank you!
Please return your survey in the self-addressed return envelope.

Physician Pre-Dissemination Survey

AMERICAN ACADEMY OF NEUROLOGY INSTITUTE

Use of Complementary and Alternative Treatments in Multiple Sclerosis

Welcome to our survey. We are interested in learning more about your attitudes and practice regarding the use of complementary and alternative medicine (CAM) in multiple sclerosis (MS). This survey should take no more than 5–10 minutes to complete. We appreciate your time and effort.

Unless otherwise instructed, please select one (1) response that best reflects your answer.

For questions, please contact:

Pushpa Narayanaswami, MBBS, DM, FAAN
Neurology TCC-8, BIDMC
330 Brookline Avenue
Boston, MA 02215
Phone: (617)-667-8130
Fax: (617)-667-3175
Email: pnarayan@bidmc.harvard.edu

Before we begin, we have a question about your use of the Internet.

1. How often are you on the Internet?
   - ☐ I rarely use a computer or the Internet
   - ☐ I use the Internet once a week
   - ☐ I use the Internet several times per week
   - ☐ I use the Internet every day

The National Center for Complementary and Alternative Medicine (NCCAM) defines complementary and alternative medicine (CAM) as medical practices and products that are not generally considered part of conventional medicine.

- ***Conventional medicine*** is medicine practiced by medical doctors and health professionals such as physical therapists, psychologists, and registered nurses. Examples of conventional medicine are vaccines, prescriptions, and surgery.
- ***Complementary medicine*** is non-conventional medicine practiced **together with** conventional medicine. An example is using acupuncture **in addition to** medication to help lessen pain.
- ***Alternative medicine*** refers to use of non-conventional medicine practiced **instead of** conventional medicine. An example of “non-conventional” therapy is using an herbal supplement such as ginkgo biloba for memory.

2. Do you routinely discuss CAM therapies with your patients with MS?
   - ☐ Yes
   - ☐ No

3. What are some common CAM therapies **your patients use** to treat or complement medical treatment of their MS? ***Please check all that apply.***

- ☐ Acupuncture
- ☐ Amalgam replacement
- ☐ Bee venom (sting)
- ☐ Biofeedback
- ☐ Cannabis extract (e.g., Cesamet, Marinol)
- ☐ Carnitine
- ☐ Chelation
- ☐ Chiropractic care
- ☐ Creatine
- ☐ Ginkgo biloba
- ☐ Glucosamine
- ☐ Hippotherapy (therapeutic horseback riding)
- ☐ Hyperbaric oxygen
- ☐ Inosine
- ☐ Linoleic acid
- ☐ Lofepramine
- ☐ Low-dose naltrexone
- ☐ Magnetic therapy
- ☐ Massage therapy
- ☐ Mindfulness
- ☐ Music therapy
- ☐ Naturopathic medicine
- ☐ Neural therapy
- ☐ Omega-3 fatty acid supplementation (e.g., fish oil)
- ☐ Padma 28
- ☐ Phenylalanine
- ☐ Progressive muscle relaxation
- ☐ Reflexology
- ☐ Smoking marijuana
- ☐ Tai chi
- ☐ Threonine
- ☐ Transdermal histamine (e.g., Prokarin patch)
- ☐ Yoga
- ☐ Other (please describe):
  ____
  ____
  ____
- ☐ I don’t know

4. Have ***you ever prescribed or recommended*** any of these CAM therapies for your patients with MS? ***Please check all that apply*** *or select "No" if you have never prescribed/recommended any CAM therapies.*

☐ **No, I have never prescribed or recommended any CAM therapies.**

- ☐ Acupuncture
- ☐ Amalgam replacement
- ☐ Bee venom (sting)
- ☐ Biofeedback
- ☐ Cannabis extract (e.g., Cesamet, Marinol)
- ☐ Carnitine
- ☐ Chelation
- ☐ Chiropractic care
- ☐ Creatine
- ☐ Ginkgo biloba
- ☐ Glucosamine
- ☐ Hippotherapy (therapeutic horseback riding)
- ☐ Hyperbaric oxygen
- ☐ Inosine
- ☐ Linoleic acid
- ☐ Lofepramine
- ☐ Low-dose naltrexone
- ☐ Magnetic therapy
- ☐ Massage therapy
- ☐ Mindfulness
- ☐ Music therapy
- ☐ Naturopathic medicine
- ☐ Neural therapy
- ☐ Omega-3 fatty acid supplementation (e.g., fish oil)
- ☐ Padma 28
- ☐ Phenylalanine
- ☐ Progressive muscle relaxation
- ☐ Reflexology
- ☐ Smoking marijuana
- ☐ Tai chi
- ☐ Threonine
- ☐ Transdermal histamine (e.g., Prokarin patch)
- ☐ Yoga
- ☐ Other (please describe):
  ____
  ____
  ____

The next set of questions is about specific CAM therapies. **Please select one (1) response that best reflects the extent to which you agree or disagree with each of the statements below.**

	**Agree**	**Somewhat agree**	**Somewhat disagree**	**Disagree**	**Not sure**
5. Magnetic therapy is effective for treating fatigue in people with MS.	☐	☐	☐	☐	☐
6. Taking oral cannabis extract is effective for treating tremors in people with MS.	☐	☐	☐	☐	☐
7. Smoking marijuana (cannabis) is effective for treating balance, posture, or pain in people with MS.	☐	☐	☐	☐	☐
8. Taking ginkgo biloba orally is effective for improving memory in people with MS.	☐	☐	☐	☐	☐
9. Hyperbaric oxygen is effective for improving symptoms in people with MS.	☐	☐	☐	☐	☐
10. Bee sting therapy is effective for reducing MRI lesions in people with MS.	☐	☐	☐	☐	☐

11. Do you ***plan to initiate discussions*** *about* any of these CAM therapies with your patients with MS? ***Please check all that apply*** *or select "No" if you do not plan on discussing any CAM therapies.*

☐ **No, I do not plan to initiate any discussions about any CAM therapies with my patients with MS.**

- ☐ Acupuncture
- ☐ Amalgam replacement
- ☐ Bee venom (sting)
- ☐ Biofeedback
- ☐ Cannabis extract (e.g., Cesamet, Marinol)
- ☐ Carnitine
- ☐ Chelation
- ☐ Chiropractic care
- ☐ Creatine
- ☐ Ginkgo biloba
- ☐ Glucosamine
- ☐ Hippotherapy (therapeutic horseback riding)
- ☐ Hyperbaric oxygen
- ☐ Inosine
- ☐ Linoleic acid
- ☐ Lofepramine
- ☐ Low-dose naltrexone
- ☐ Magnetic therapy
- ☐ Massage therapy
- ☐ Mindfulness
- ☐ Music therapy
- ☐ Naturopathic medicine
- ☐ Neural therapy
- ☐ Omega-3 fatty acid supplementation (e.g., fish oil)
- ☐ Padma 28
- ☐ Phenylalanine
- ☐ Progressive muscle relaxation
- ☐ Reflexology
- ☐ Smoking marijuana
- ☐ Tai chi
- ☐ Threonine
- ☐ Transdermal histamine (e.g., Prokarin patch)
- ☐ Yoga
- ☐ Other (please describe): ___________________________________ ___________________________________

12. Do you think any of the CAM therapies listed below ***are useful*** for treating MS? ***Please check all that apply*** *or select "No" if you do not think any CAM therapies are useful for treating MS.*

☐ **No, I do not think any of the CAM therapies listed below are useful for treating MS.**

☐ Acupuncture
☐ Amalgam replacement
☐ Bee venom (sting)
☐ Biofeedback
☐ Cannabis extract (e.g., Cesamet, Marinol)
☐ Carnitine
☐ Chelation
☐ Chiropractic care
☐ Creatine
☐ Ginkgo biloba
☐ Glucosamine
☐ Hippotherapy (therapeutic horseback riding)
☐ Hyperbaric oxygen
☐ Inosine
☐ Linoleic acid
☐ Lofepramine
☐ Low-dose naltrexone
☐ Magnetic therapy
☐ Massage therapy
☐ Mindfulness
☐ Music therapy
☐ Naturopathic medicine
☐ Neural therapy
☐ Omega-3 fatty acid supplementation (e.g., fish oil)
☐ Padma 28
☐ Phenylalanine
☐ Progressive muscle relaxation
☐ Reflexology
☐ Smoking marijuana
☐ Tai chi
☐ Threonine
☐ Transdermal histamine (e.g., Prokarin patch)
☐ Yoga
☐ Other (please describe):
____
____
____

A **clinical practice guideline** is a document that helps guide the decisions made by health care providers. Guidelines assess tests for diagnosing or therapies for treating a specific disease. Guidelines from the American Academy of Neurology (AAN) are developed by neurologists and other experts. The guidelines are based on a careful review of all available scientific studies for a specific brain disease.

13. Did you know that the AAN has developed a guideline about the use of CAM therapies in MS?
   ☐ Yes
   ☐ No

14. Do you discuss AAN guideline recommendations with your patients with MS?
   ☐ Yes → (go to 14a)
   ☐ No → **Please continue with Question 15 on Page 8.**

   14a. When you speak with patients about guideline-related information, do you use or distribute guideline summaries or tools for patients and their families?
      ☐ Yes
      ☐ No

The final set of questions is about you and your practice.

15. How old are you?
    ________ years old

16. What is your gender?
    ☐ Female
    ☐ Male
    ☐ Prefer not to answer

17. Do you consider yourself Hispanic or Latino/a?
    ☐ Yes, Hispanic or Latino/a
    ☐ No

18. What is your race? ***Please check all that apply.***
    ☐ American Indian or Native American
    ☐ Asian
    ☐ Black or African American
    ☐ Hawaiian or other Pacific Islander
    ☐ White
    ☐ Other (please specify):
    ____

19. What is your practice focus?
    ☐ Solo practice
    ☐ Neurology group
    ☐ Multispecialty group
    ☐ University-based group
    ☐ Staff-model HMO
    ☐ Government hospital or clinic
    ☐ Other public/private hospital clinic (please specify):________________
    ☐ No clinical practice

20. How many years have you been in practice?
    ☐ 0–5 years
    ☐ 6–10 years
    ☐ 11–15 years
    ☐ 16–20 years
    ☐ 21–25 years
    ☐ 26–30 years
    ☐ 31+ years

21. How many patients are you presently treating for their symptoms of MS?
    ☐ 0–10 patients with MS
    ☐ 11–20 patients with MS
    ☐ 21–30 patients with MS
    ☐ 31–40 patients with MS
    ☐ 41–50 patients with MS
    ☐ 51+ patients with MS

Thank you for your feedback!
Please return your survey in the self-addressed return envelope.

Physician Post-Dissemination Survey 1

Use of Complementary and Alternative Treatments in Multiple Sclerosis

Welcome to our survey. We are interested in learning more about your attitudes and practice regarding the use of complementary and alternative medicine (CAM) in multiple sclerosis (MS). This survey should take no more than 5–10 minutes to complete. We appreciate your time and effort.

Unless otherwise instructed, please select one (1) response that best reflects your answer.

For questions, please contact:
Pushpa Narayanaswami, MBBS, DM, FAAN
Neurology TCC-8, BIDMC
330 Brookline Avenue
Boston, MA 02215
Phone: (617)-667-8130
Fax: (617)-667-3175
Email: pnarayan@bidmc.harvard.edu

Before we begin, we have a question about your use of the Internet.

1. How often are you on the Internet?
   - ☐ I rarely use a computer or the Internet
   - ☐ I use the Internet once a week
   - ☐ I use the Internet several times per week
   - ☐ I use the Internet every day

The National Center for Complementary and Alternative Medicine (NCCAM) defines complementary and alternative medicine (CAM) as medical practices and products that are not generally considered part of conventional medicine.

- ***Conventional medicine*** is medicine practiced by medical doctors and health professionals such as physical therapists, psychologists, and registered nurses. Examples of conventional medicine are vaccines, prescriptions, and surgery.
- ***Complementary medicine*** is non-conventional medicine practiced **together with** conventional medicine. An example is using acupuncture **in addition to** medication to help lessen pain.
- ***Alternative medicine*** refers to use of non-conventional medicine practiced **instead of** conventional medicine. An example of "non-conventional" therapy is using an herbal supplement such as ginkgo biloba for memory.

2. Do you routinely discuss CAM therapies with your patients with MS?
   - ☐ Yes
   - ☐ No

3. What are some common CAM therapies **your patients use** to treat or complement medical treatment of their MS? ***Please check all that apply.***

- ☐ Acupuncture
- ☐ Amalgam replacement
- ☐ Bee venom (sting)
- ☐ Biofeedback
- ☐ Cannabis extract (e.g., Cesamet, Marinol)
- ☐ Carnitine
- ☐ Chelation
- ☐ Chiropractic care
- ☐ Creatine
- ☐ Ginkgo biloba
- ☐ Glucosamine
- ☐ Hippotherapy (therapeutic horseback riding)
- ☐ Hyperbaric oxygen
- ☐ Inosine
- ☐ Linoleic acid
- ☐ Lofepramine
- ☐ Low-dose naltrexone
- ☐ Magnetic therapy
- ☐ Massage therapy
- ☐ Mindfulness
- ☐ Music therapy
- ☐ Naturopathic medicine
- ☐ Neural therapy
- ☐ Omega-3 fatty acid supplementation (e.g., fish oil)
- ☐ Padma 28
- ☐ Phenylalanine
- ☐ Progressive muscle relaxation
- ☐ Reflexology
- ☐ Smoking marijuana
- ☐ Tai chi
- ☐ Threonine
- ☐ Transdermal histamine (e.g., Prokarin patch)
- ☐ Yoga
- ☐ Other (please describe):
  ____
  ____
  ____
- ☐ I don’t know

4. Have ***you ever prescribed or recommended*** any of these CAM therapies for your patients with MS? ***Please check all that apply*** *or select "No" if you have never prescribed/recommended any CAM therapies.*

☐ **No, I have never prescribed or recommended any CAM therapies.**

- ☐ Acupuncture
- ☐ Amalgam replacement
- ☐ Bee venom (sting)
- ☐ Biofeedback
- ☐ Cannabis extract (e.g., Cesamet, Marinol)
- ☐ Carnitine
- ☐ Chelation
- ☐ Chiropractic care
- ☐ Creatine
- ☐ Ginkgo biloba
- ☐ Glucosamine
- ☐ Hippotherapy (therapeutic horseback riding)
- ☐ Hyperbaric oxygen
- ☐ Inosine
- ☐ Linoleic acid
- ☐ Lofepramine
- ☐ Low-dose naltrexone
- ☐ Magnetic therapy
- ☐ Massage therapy
- ☐ Mindfulness
- ☐ Music therapy
- ☐ Naturopathic medicine
- ☐ Neural therapy
- ☐ Omega-3 fatty acid supplementation (e.g., fish oil)
- ☐ Padma 28
- ☐ Phenylalanine
- ☐ Progressive muscle relaxation
- ☐ Reflexology
- ☐ Smoking marijuana
- ☐ Tai chi
- ☐ Threonine
- ☐ Transdermal histamine (e.g., Prokarin patch)
- ☐ Yoga
- ☐ Other (please describe): ______________________________ ______________________________

The next set of questions is about specific CAM therapies. **Please select one (1) response that best reflects the extent to which you agree or disagree with each of the statements below.**

	**Agree**	**Somewhat agree**	**Somewhat disagree**	**Disagree**	**Not sure**
5. Magnetic therapy is effective for treating fatigue in people with MS.	☐	☐	☐	☐	☐
6. Taking oral cannabis extract is effective for treating tremors in people with MS.	☐	☐	☐	☐	☐
7. Smoking marijuana (cannabis) is effective for treating balance, posture, or pain in people with MS.	☐	☐	☐	☐	☐
8. Taking ginkgo biloba orally is effective for improving memory in people with MS.	☐	☐	☐	☐	☐
9. Hyperbaric oxygen is effective for improving symptoms in people with MS.	☐	☐	☐	☐	☐
10. Bee sting therapy is effective for reducing MRI lesions in people with MS.	☐	☐	☐	☐	☐

11. Do you think any of the CAM therapies listed below ***are useful*** for treating MS? ***Please check all that apply*** *or select "No" if you do not think any CAM therapies are useful for treating MS.*

☐ **No, I do not think any of the CAM therapies listed below are useful for treating MS.**

☐ Acupuncture
☐ Amalgam replacement
☐ Bee venom (sting)
☐ Biofeedback
☐ Cannabis extract (e.g., Cesamet, Marinol)
☐ Carnitine
☐ Chelation
☐ Chiropractic care
☐ Creatine
☐ Ginkgo biloba
☐ Glucosamine
☐ Hippotherapy (therapeutic horseback riding)
☐ Hyperbaric oxygen
☐ Inosine
☐ Linoleic acid
☐ Lofepramine
☐ Low-dose naltrexone
☐ Magnetic therapy
☐ Massage therapy
☐ Mindfulness
☐ Music therapy
☐ Naturopathic medicine
☐ Neural therapy
☐ Omega-3 fatty acid supplementation (e.g., fish oil)
☐ Padma 28
☐ Phenylalanine
☐ Progressive muscle relaxation
☐ Reflexology
☐ Smoking marijuana
☐ Tai chi
☐ Threonine
☐ Transdermal histamine (e.g., Prokarin patch)
☐ Yoga
☐ Other (please describe):
____
____

A **clinical practice guideline** is a document that helps guide the decisions made by health care providers. Guidelines assess tests for diagnosing or therapies for treating a specific disease. Guidelines from the American Academy of Neurology (AAN) are developed by neurologists and other experts. The guidelines are based on a careful review of all available scientific studies for a specific brain disease.

12. Did you know that the AAN has developed a guideline about the use of CAM therapies in MS?
   ☐ Yes
   ☐ No ——▶ **Please continue with Question 14 on page 7.**

12a. How did you hear about the guideline?
Please check all that apply and identify the source.
- ☐ AAN website
- ☐ Colleague
- ☐ Email sent to me
- ☐ Google+
- ☐ Facebook
- ☐ LinkedIn
- ☐ Medical society ____
- ☐ My patient asked about it
- ☐ *Neurology*® journal
- ☐ *Neurology Now*®
- ☐ *Neurology Today*®
- ☐ News media source (please identify): ____________________________
- ☐ Pinterest
- ☐ Twitter
- ☐ Website (please identify): ___________________________________
- ☐ Your institution (please identify): ______________________________
- ☐ YouTube
- ☐ Other (please describe):_______________________________________

13. Have you read the AAN guideline or physician/patient summary on CAM use in MS?
   ☐ Yes
   ☐ No ——▶ **Please continue with Question 14 on page 7.**

13a. What sources did you use to read or access the guideline?
Please check all that apply.
- ☐ AAN website
- ☐ E-mail sent to me
- ☐ Google+
- ☐ Facebook
- ☐ LinkedIn
- ☐ *Neurology* journal
- ☐ *Neurology Now*
- ☐ *Neurology Today*
- ☐ Pinterest
- ☐ Twitter
- ☐ Website (please identify): ______________
- ☐ YouTube
- ☐ Other (please describe): ______________________________________

14. Do you ***plan to initiate discussions*** *about* any of these CAM therapies with your patients with MS? ***Please check all that apply*** *or select "No" if you do not plan on discussing any CAM therapies.*

- ☐ **No, I do not plan to initiate any discussions about any CAM therapies with my patients with MS.**

- ☐ Acupuncture
- ☐ Amalgam replacement
- ☐ Bee venom (sting)
- ☐ Biofeedback
- ☐ Cannabis extract (e.g., Cesamet, Marinol)
- ☐ Carnitine
- ☐ Chelation
- ☐ Chiropractic care
- ☐ Creatine
- ☐ Ginkgo biloba
- ☐ Glucosamine
- ☐ Hippotherapy (therapeutic horseback riding)
- ☐ Hyperbaric oxygen
- ☐ Inosine
- ☐ Linoleic acid
- ☐ Lofepramine
- ☐ Low-dose naltrexone
- ☐ Magnetic therapy
- ☐ Massage therapy
- ☐ Mindfulness
- ☐ Music therapy
- ☐ Naturopathic medicine
- ☐ Neural therapy
- ☐ Omega-3 fatty acid supplementation (e.g., fish oil)
- ☐ Padma 28
- ☐ Phenylalanine
- ☐ Progressive muscle relaxation
- ☐ Reflexology
- ☐ Smoking marijuana
- ☐ Tai chi
- ☐ Threonine
- ☐ Transdermal histamine (e.g., Prokarin patch)
- ☐ Yoga
- ☐ Other (please describe): ___________________________________ ___________________________________

15. Since *March 2014*, have you advised patients ***to start using or stop using*** any of these CAM therapies for MS? *Please check all that apply for both questions.*

	I have advised patients to ***start using*** this therapy.	I have advised patients to ***stop using*** this therapy.
Acupuncture	☐	☐
Amalgam replacement	☐	☐
Bee venom (sting)	☐	☐
Biofeedback	☐	☐
Cannabis extract (e.g., Cesamet, Marinol)	☐	☐
Carnitine	☐	☐
Chelation	☐	☐
Chiropractic care	☐	☐
Creatine	☐	☐
Ginkgo biloba	☐	☐
Glucosamine	☐	☐
Hippotherapy (therapeutic horseback riding)	☐	☐
Hyperbaric oxygen	☐	☐
Inosine	☐	☐
Linoleic acid	☐	☐
Lofepramine	☐	☐
Low-dose naltrexone	☐	☐
Magnetic therapy	☐	☐
Massage therapy	☐	☐
Mindfulness	☐	☐
Music therapy	☐	☐
Naturopathic medicine	☐	☐
Neural therapy	☐	☐
Omega-3 fatty acid supplementation (e.g., fish oil)	☐	☐
Padma 28	☐	☐
Phenylalanine	☐	☐
Progressive muscle relaxation	☐	☐
Reflexology	☐	☐
Smoking marijuana	☐	☐
Tai chi	☐	☐
Threonine	☐	☐
Transdermal histamine (e.g., Prokarin patch)	☐	☐
Yoga	☐	☐
Other (please describe): ___________________________ ___________________________	☐	☐

16. Do you discuss AAN guideline recommendations with your patients with MS?
    - ☐ Yes
    - ☐ No → **Please continue with Question 17.**

16a. When you speak with patients about guideline-related information, do you use or distribute guideline summaries or tools for patients and their families?
- ☐ Yes
- ☐ No

17. Because of new information gained from AAN guidelines on the use of CAM in MS, have you changed your practice regarding discussing CAM therapies with your patients with MS?
    - ☐ Yes, I have changed my practice → **Please go to Question 17a.**
    - ☐ No, I do not do anything different → **Please go to Question 18 on Page 10.**

17a. Please describe how you have changed your practice.
____
____
____

The final set of questions is about you and your practice.

18. How old are you?
    ________ years old

19. What is your gender?
    ☐ Female
    ☐ Male
    ☐ Prefer not to answer

20. Do you consider yourself Hispanic or Latino/a?
    ☐ Yes, Hispanic or Latino/a
    ☐ No

21. What is your race? ***Please check all that apply.***
    ☐ American Indian or Native American
    ☐ Asian
    ☐ Black or African American
    ☐ Hawaiian or other Pacific Islander
    ☐ White
    ☐ Other (please specify):
    ____

22. What is your practice focus?
    ☐ Solo practice
    ☐ Neurology group
    ☐ Multispecialty group
    ☐ University-based group
    ☐ Staff-model HMO
    ☐ Government hospital or clinic
    ☐ Other public/private hospital clinic (please specify):
    ____
    ☐ No clinical practice

23. How many years have you been in practice?
    ☐ 0–5 years
    ☐ 6–10 years
    ☐ 11–15 years
    ☐ 16–20 years
    ☐ 21–25 years
    ☐ 26–30 years
    ☐ 31+ years

24. How many patients are you presently treating for their symptoms of MS?
    ☐ 0–10 patients with MS
    ☐ 11–20 patients with MS
    ☐ 21–30 patients with MS
    ☐ 31–40 patients with MS
    ☐ 41–50 patients with MS
    ☐ 51+ patients with MS

Thank you for your feedback!
Please return your survey in the self-addressed return envelope.

Physician Post-Dissemination Survey 2

Use of Complementary and Alternative Treatments in Multiple Sclerosis

Welcome to our survey. We are interested in learning more about your attitudes and practice regarding the use of complementary and alternative medicine (CAM) in multiple sclerosis (MS). This survey should take no more than 5–10 minutes to complete. We appreciate your time and effort.

Unless otherwise instructed, please select one (1) response that best reflects your answer.

For questions, please contact:

Pushpa Narayanaswami, MBBS, DM, FAAN
Neurology TCC-8, BIDMC
330 Brookline Avenue
Boston, MA 02215
Phone: (617)-667-8130
Fax: (617)-667-3175
Email: pnarayan@bidmc.harvard.edu

Before we begin, we have a question about your use of the Internet.

1. How often are you on the Internet?
   - ☐ I rarely use a computer or the Internet
   - ☐ I use the Internet once a week
   - ☐ I use the Internet several times per week
   - ☐ I use the Internet every day

The National Center for Complementary and Alternative Medicine (NCCAM) defines complementary and alternative medicine (CAM) as medical practices and products that are not generally considered part of conventional medicine.

- ***Conventional medicine*** is medicine practiced by medical doctors and health professionals such as physical therapists, psychologists, and registered nurses. Examples of conventional medicine are vaccines, prescriptions, and surgery.
- ***Complementary medicine*** is non-conventional medicine practiced **together with** conventional medicine. An example is using acupuncture **in addition to** medication to help lessen pain.
- ***Alternative medicine*** refers to use of non-conventional medicine practiced **instead of** conventional medicine. An example of “non-conventional” therapy is using an herbal supplement such as ginkgo biloba for memory.

2. Do you routinely discuss CAM therapies with your patients with MS?
   - ☐ Yes
   - ☐ No

3. What are some common CAM therapies **your patients use** to treat or complement medical treatment of their MS? ***Please check all that apply.***

- ☐ Acupuncture
- ☐ Amalgam replacement
- ☐ Bee venom (sting)
- ☐ Biofeedback
- ☐ Cannabis extract (e.g., Cesamet, Marinol)
- ☐ Carnitine
- ☐ Chelation
- ☐ Chiropractic care
- ☐ Creatine
- ☐ Ginkgo biloba
- ☐ Glucosamine
- ☐ Hippotherapy (therapeutic horseback riding)
- ☐ Hyperbaric oxygen
- ☐ Inosine
- ☐ Linoleic acid
- ☐ Lofepramine
- ☐ Low-dose naltrexone
- ☐ Magnetic therapy
- ☐ Massage therapy
- ☐ Mindfulness
- ☐ Music therapy
- ☐ Naturopathic medicine
- ☐ Neural therapy
- ☐ Omega-3 fatty acid supplementation (e.g., fish oil)
- ☐ Padma 28
- ☐ Phenylalanine
- ☐ Progressive muscle relaxation
- ☐ Reflexology
- ☐ Smoking marijuana
- ☐ Tai chi
- ☐ Threonine
- ☐ Transdermal histamine (e.g., Prokarin patch)
- ☐ Yoga
- ☐ Other (please describe):
  ____
  ____
  ____
- ☐ I don’t know

4. Have ***you ever prescribed or recommended*** any of these CAM therapies for your patients with MS? ***Please check all that apply*** *or select "No" if you have never prescribed/recommended any CAM therapies.*

☐ **No, I have never prescribed or recommended any CAM therapies.**

☐ Acupuncture
☐ Amalgam replacement
☐ Bee venom (sting)
☐ Biofeedback
☐ Cannabis extract (e.g., Cesamet, Marinol)
☐ Carnitine
☐ Chelation
☐ Chiropractic care
☐ Creatine
☐ Ginkgo biloba
☐ Glucosamine
☐ Hippotherapy (therapeutic horseback riding)
☐ Hyperbaric oxygen
☐ Inosine
☐ Linoleic acid
☐ Lofepramine
☐ Low-dose naltrexone
☐ Magnetic therapy
☐ Massage therapy
☐ Mindfulness
☐ Music therapy
☐ Naturopathic medicine
☐ Neural therapy
☐ Omega-3 fatty acid supplementation (e.g., fish oil)
☐ Padma 28
☐ Phenylalanine
☐ Progressive muscle relaxation
☐ Reflexology
☐ Smoking marijuana
☐ Tai chi
☐ Threonine
☐ Transdermal histamine (e.g., Prokarin patch)
☐ Yoga
☐ Other (please describe): ___________________________________ ___________________________________

The next set of questions is about specific CAM therapies. **Please select one (1) response that best reflects the extent to which you agree or disagree with each of the statements below.**

	**Agree**	**Somewhat agree**	**Somewhat disagree**	**Disagree**	**Not sure**
5. Magnetic therapy is effective for treating fatigue in people with MS.	☐	☐	☐	☐	☐
6. Taking oral cannabis extract is effective for treating tremors in people with MS.	☐	☐	☐	☐	☐
7. Smoking marijuana (cannabis) is effective for treating balance, posture, or pain in people with MS.	☐	☐	☐	☐	☐
8. Taking ginkgo biloba orally is effective for improving memory in people with MS.	☐	☐	☐	☐	☐
9. Hyperbaric oxygen is effective for improving symptoms in people with MS.	☐	☐	☐	☐	☐
10. Bee sting therapy is effective for reducing MRI lesions in people with MS.	☐	☐	☐	☐	☐

11. Do you think any of the CAM therapies listed below ***are useful*** for treating MS? ***Please check all that apply*** *or select "No" if you do not think any CAM therapies are useful for treating MS.*

☐ **No, I do not think any of the CAM therapies listed below are useful for treating MS.**

- ☐ Acupuncture
- ☐ Amalgam replacement
- ☐ Bee venom (sting)
- ☐ Biofeedback
- ☐ Cannabis extract (e.g., Cesamet, Marinol)
- ☐ Carnitine
- ☐ Chelation
- ☐ Chiropractic care
- ☐ Creatine
- ☐ Ginkgo biloba
- ☐ Glucosamine
- ☐ Hippotherapy (therapeutic horseback riding)
- ☐ Hyperbaric oxygen
- ☐ Inosine
- ☐ Linoleic acid
- ☐ Lofepramine
- ☐ Low-dose naltrexone
- ☐ Magnetic therapy
- ☐ Massage therapy
- ☐ Mindfulness
- ☐ Music therapy
- ☐ Naturopathic medicine
- ☐ Neural therapy
- ☐ Omega-3 fatty acid supplementation (e.g., fish oil)
- ☐ Padma 28
- ☐ Phenylalanine
- ☐ Progressive muscle relaxation
- ☐ Reflexology
- ☐ Smoking marijuana
- ☐ Tai chi
- ☐ Threonine
- ☐ Transdermal histamine (e.g., Prokarin patch)
- ☐ Yoga
- ☐ Other (please describe):
  ____
  ____

A **clinical practice guideline** is a document that helps guide the decisions made by health care providers. Guidelines assess tests for diagnosing or therapies for treating a specific disease. Guidelines from the American Academy of Neurology (AAN) are developed by neurologists and other experts. The guidelines are based on a careful review of all available scientific studies for a specific brain disease.

12. Did you know that the AAN has developed a guideline about the use of CAM therapies in MS?
    - ☐ Yes
    - ☐ No → **Please continue with Question 14 on page 7.**

12a. How did you hear about the guideline?
Please check all that apply and identify the source.
- ☐ AAN website
- ☐ Colleague
- ☐ Email sent to me
- ☐ Google+
- ☐ Facebook
- ☐ LinkedIn
- ☐ Medical society ______________________________
- ☐ My patient asked about it
- ☐ *Neurology®* journal
- ☐ *Neurology Now®*
- ☐ *Neurology Today®*
- ☐ News media source (please identify): ______________________
- ☐ Pinterest
- ☐ Twitter
- ☐ Website (please identify): ______________________
- ☐ Your institution (please identify): ______________________
- ☐ YouTube
- ☐ Other (please describe):______________________

13. Have you read the AAN guideline or physician/patient summary on CAM use in MS?
    - ☐ Yes
    - ☐ No → **Please continue with Question 14 on page 7.**

13a. What sources did you use to read or access the guideline?
Please check all that apply.
- ☐ AAN website
- ☐ E-mail sent to me
- ☐ Google+
- ☐ Facebook
- ☐ LinkedIn
- ☐ *Neurology* journal
- ☐ *Neurology Now*
- ☐ *Neurology Today*
- ☐ Pinterest
- ☐ Twitter
- ☐ Website (please identify): ______________
- ☐ YouTube
- ☐ Other (please describe): ______________________

14. Do you ***plan to initiate discussions*** *about* any of these CAM therapies with your patients with MS? ***Please check all that apply*** *or select "No" if you do not plan on discussing any CAM therapies.*

□ **No, I do not plan to initiate any discussions about any CAM therapies with my patients with MS.**

- ☐ Acupuncture
- ☐ Amalgam replacement
- ☐ Bee venom (sting)
- ☐ Biofeedback
- ☐ Cannabis extract (e.g., Cesamet, Marinol)
- ☐ Carnitine
- ☐ Chelation
- ☐ Chiropractic care
- ☐ Creatine
- ☐ Ginkgo biloba
- ☐ Glucosamine
- ☐ Hippotherapy (therapeutic horseback riding)
- ☐ Hyperbaric oxygen
- ☐ Inosine
- ☐ Linoleic acid
- ☐ Lofepramine
- ☐ Low-dose naltrexone
- ☐ Magnetic therapy
- ☐ Massage therapy
- ☐ Mindfulness
- ☐ Music therapy
- ☐ Naturopathic medicine
- ☐ Neural therapy
- ☐ Omega-3 fatty acid supplementation (e.g., fish oil)
- ☐ Padma 28
- ☐ Phenylalanine
- ☐ Progressive muscle relaxation
- ☐ Reflexology
- ☐ Smoking marijuana
- ☐ Tai chi
- ☐ Threonine
- ☐ Transdermal histamine (e.g., Prokarin patch)
- ☐ Yoga
- ☐ Other (please describe): ________________________________ ________________________________

15. Since *late June 2014*, have you advised patients ***to start using or stop using*** any of these CAM therapies for MS? *Please check all that apply for both questions.*

	I have advised patients to ***start using*** this therapy.	I have advised patients to ***stop using*** this therapy.
Acupuncture	☐	☐
Amalgam replacement	☐	☐
Bee venom (sting)	☐	☐
Biofeedback	☐	☐
Cannabis extract (e.g., Cesamet, Marinol)	☐	☐
Carnitine	☐	☐
Chelation	☐	☐
Chiropractic care	☐	☐
Creatine	☐	☐
Ginkgo biloba	☐	☐
Glucosamine	☐	☐
Hippotherapy (therapeutic horseback riding)	☐	☐
Hyperbaric oxygen	☐	☐
Inosine	☐	☐
Linoleic acid	☐	☐
Lofepramine	☐	☐
Low-dose naltrexone	☐	☐
Magnetic therapy	☐	☐
Massage therapy	☐	☐
Mindfulness	☐	☐
Music therapy	☐	☐
Naturopathic medicine	☐	☐
Neural therapy	☐	☐
Omega-3 fatty acid supplementation (e.g., fish oil)	☐	☐
Padma 28	☐	☐
Phenylalanine	☐	☐
Progressive muscle relaxation	☐	☐
Reflexology	☐	☐
Smoking marijuana	☐	☐
Tai chi	☐	☐
Threonine	☐	☐
Transdermal histamine (e.g., Prokarin patch)	☐	☐
Yoga	☐	☐
Other (please describe): ____________________________ ____________________________	☐	☐

16. Do you discuss AAN guideline recommendations with your patients with MS?
   - ☐ Yes → (go to 16a)
   - ☐ No → **Please continue with Question 17.**

16a. When you speak with patients about guideline-related information, do you use or distribute guideline summaries or tools for patients and their families?
   - ☐ Yes
   - ☐ No

17. Because of new information gained from AAN guidelines on the use of CAM in MS, have you changed your practice regarding discussing CAM therapies with your patients with MS?
   - ☐ Yes, I have changed my practice → **Please go to Question 17a.**
   - ☐ No, I do not do anything different → **Please go to Question 18 on Page 10.**

17a. Please describe how you have changed your practice.
____
____
____

The final set of questions is about you and your practice.

18. How old are you?
    ________ years old

19. What is your gender?
    - ☐ Female
    - ☐ Male
    - ☐ Prefer not to answer

20. Do you consider yourself Hispanic or Latino/a?
    - ☐ Yes, Hispanic or Latino/a
    - ☐ No

21. What is your race? ***Please check all that apply.***
    - ☐ American Indian or Native American
    - ☐ Asian
    - ☐ Black or African American
    - ☐ Hawaiian or other Pacific Islander
    - ☐ White
    - ☐ Other (please specify):
      ____

22. What is your practice focus?
    - ☐ Solo practice
    - ☐ Neurology group
    - ☐ Multispecialty group
    - ☐ University-based group
    - ☐ Staff-model HMO
    - ☐ Government hospital or clinic
    - ☐ Other public/private hospital clinic (please specify):
      ____
    - ☐ No clinical practice

23. How many years have you been in practice?
    - ☐ 0–5 years
    - ☐ 6–10 years
    - ☐ 11–15 years
    - ☐ 16–20 years
    - ☐ 21–25 years
    - ☐ 26–30 years
    - ☐ 31+ years

24. How many patients are you presently treating for their symptoms of MS?
    - ☐ 0–10 patients with MS
    - ☐ 11–20 patients with MS
    - ☐ 21–30 patients with MS
    - ☐ 31–40 patients with MS
    - ☐ 41–50 patients with MS
    - ☐ 51+ patients with MS

Thank you for your feedback!
Please return your survey in the self-addressed return envelope.

Physician Post-Dissemination Survey 3

AMERICAN ACADEMY OF NEUROLOGY INSTITUTE

Use of Complementary and Alternative Treatments in Multiple Sclerosis

Welcome to our survey. We are interested in learning more about your attitudes and practice regarding the use of complementary and alternative medicine (CAM) in multiple sclerosis (MS). This survey should take no more than 5–10 minutes to complete. We appreciate your time and effort.

Unless otherwise instructed, please select one (1) response that best reflects your answer.

For questions, please contact:

Pushpa Narayanaswami, MBBS, DM, FAAN
Neurology TCC-8, BIDMC
330 Brookline Avenue
Boston, MA 02215
Phone: (617)-667-8130
Fax: (617)-667-3175
Email: pnarayan@bidmc.harvard.edu

Before we begin, we have a question about your use of the Internet.

1. How often are you on the Internet?
   - ☐ I rarely use a computer or the Internet
   - ☐ I use the Internet once a week
   - ☐ I use the Internet several times per week
   - ☐ I use the Internet every day

The National Center for Complementary and Alternative Medicine (NCCAM) defines complementary and alternative medicine (CAM) as medical practices and products that are not generally considered part of conventional medicine.

- ***Conventional medicine*** is medicine practiced by medical doctors and health professionals such as physical therapists, psychologists, and registered nurses. Examples of conventional medicine are vaccines, prescriptions, and surgery.
- ***Complementary medicine*** is non-conventional medicine practiced **together with** conventional medicine. An example is using acupuncture **in addition to** medication to help lessen pain.
- ***Alternative medicine*** refers to use of non-conventional medicine practiced **instead of** conventional medicine. An example of “non-conventional” therapy is using an herbal supplement such as ginkgo biloba for memory.

2. Do you routinely discuss CAM therapies with your patients with MS?
   - ☐ Yes
   - ☐ No

3. What are some common CAM therapies **your patients use** to treat or complement medical treatment of their MS? ***Please check all that apply.***

- ☐ Acupuncture
- ☐ Amalgam replacement
- ☐ Bee venom (sting)
- ☐ Biofeedback
- ☐ Cannabis extract (e.g., Cesamet, Marinol)
- ☐ Carnitine
- ☐ Chelation
- ☐ Chiropractic care
- ☐ Creatine
- ☐ Ginkgo biloba
- ☐ Glucosamine
- ☐ Hippotherapy (therapeutic horseback riding)
- ☐ Hyperbaric oxygen
- ☐ Inosine
- ☐ Linoleic acid
- ☐ Lofepramine
- ☐ Low-dose naltrexone
- ☐ Magnetic therapy
- ☐ Massage therapy
- ☐ Mindfulness
- ☐ Music therapy
- ☐ Naturopathic medicine
- ☐ Neural therapy
- ☐ Omega-3 fatty acid supplementation (e.g., fish oil)
- ☐ Padma 28
- ☐ Phenylalanine
- ☐ Progressive muscle relaxation
- ☐ Reflexology
- ☐ Smoking marijuana
- ☐ Tai chi
- ☐ Threonine
- ☐ Transdermal histamine (e.g., Prokarin patch)
- ☐ Yoga
- ☐ Other (please describe):
  ____
  ____
  ____
- ☐ I don’t know

4. Have ***you ever prescribed or recommended*** any of these CAM therapies for your patients with MS? ***Please check all that apply*** *or select "No" if you have never prescribed/recommended any CAM therapies.*

☐ **No, I have never prescribed or recommended any CAM therapies.**

- ☐ Acupuncture
- ☐ Amalgam replacement
- ☐ Bee venom (sting)
- ☐ Biofeedback
- ☐ Cannabis extract (e.g., Cesamet, Marinol)
- ☐ Carnitine
- ☐ Chelation
- ☐ Chiropractic care
- ☐ Creatine
- ☐ Ginkgo biloba
- ☐ Glucosamine
- ☐ Hippotherapy (therapeutic horseback riding)
- ☐ Hyperbaric oxygen
- ☐ Inosine
- ☐ Linoleic acid
- ☐ Lofepramine
- ☐ Low-dose naltrexone
- ☐ Magnetic therapy
- ☐ Massage therapy
- ☐ Mindfulness
- ☐ Music therapy
- ☐ Naturopathic medicine
- ☐ Neural therapy
- ☐ Omega-3 fatty acid supplementation (e.g., fish oil)
- ☐ Padma 28
- ☐ Phenylalanine
- ☐ Progressive muscle relaxation
- ☐ Reflexology
- ☐ Smoking marijuana
- ☐ Tai chi
- ☐ Threonine
- ☐ Transdermal histamine (e.g., Prokarin patch)
- ☐ Yoga
- ☐ Other (please describe): ____________________________________ ____________________________________

The next set of questions is about specific CAM therapies. **Please select one (1) response that best reflects the extent to which you agree or disagree with each of the statements below.**

	**Agree**	**Somewhat agree**	**Somewhat disagree**	**Disagree**	**Not sure**
5. Magnetic therapy is effective for treating fatigue in people with MS.	☐	☐	☐	☐	☐
6. Taking oral cannabis extract is effective for treating tremors in people with MS.	☐	☐	☐	☐	☐
7. Smoking marijuana (cannabis) is effective for treating balance, posture, or pain in people with MS.	☐	☐	☐	☐	☐
8. Taking ginkgo biloba orally is effective for improving memory in people with MS.	☐	☐	☐	☐	☐
9. Hyperbaric oxygen is effective for improving symptoms in people with MS.	☐	☐	☐	☐	☐
10. Bee sting therapy is effective for reducing MRI lesions in people with MS.	☐	☐	☐	☐	☐

11. Do you think any of the CAM therapies listed below ***are useful*** for treating MS? ***Please check all that apply*** *or select "No" if you do not think any CAM therapies are useful for treating MS.*

☐ **No, I do not think any of the CAM therapies listed below are useful for treating MS.**

- ☐ Acupuncture
- ☐ Amalgam replacement
- ☐ Bee venom (sting)
- ☐ Biofeedback
- ☐ Cannabis extract (e.g., Cesamet, Marinol)
- ☐ Carnitine
- ☐ Chelation
- ☐ Chiropractic care
- ☐ Creatine
- ☐ Ginkgo biloba
- ☐ Glucosamine
- ☐ Hippotherapy (therapeutic horseback riding)
- ☐ Hyperbaric oxygen
- ☐ Inosine
- ☐ Linoleic acid
- ☐ Lofepramine
- ☐ Low-dose naltrexone
- ☐ Magnetic therapy
- ☐ Massage therapy
- ☐ Mindfulness
- ☐ Music therapy
- ☐ Naturopathic medicine
- ☐ Neural therapy
- ☐ Omega-3 fatty acid supplementation (e.g., fish oil)
- ☐ Padma 28
- ☐ Phenylalanine
- ☐ Progressive muscle relaxation
- ☐ Reflexology
- ☐ Smoking marijuana
- ☐ Tai chi
- ☐ Threonine
- ☐ Transdermal histamine (e.g., Prokarin patch)
- ☐ Yoga
- ☐ Other (please describe):
  ____
  ____

A **clinical practice guideline** is a document that helps guide the decisions made by health care providers. Guidelines assess tests for diagnosing or therapies for treating a specific disease. Guidelines from the American Academy of Neurology (AAN) are developed by neurologists and other experts. The guidelines are based on a careful review of all available scientific studies for a specific brain disease.

12. Did you know that the AAN has developed a guideline about the use of CAM therapies in MS?
- ☐ Yes
- ☐ No → **Please continue with Question 14 on page 7.**

12a. How did you hear about the guideline?
Please check all that apply and identify the source.
- ☐ AAN website
- ☐ Colleague
- ☐ Email sent to me
- ☐ Google+
- ☐ Facebook
- ☐ LinkedIn
- ☐ Medical society ______________________________
- ☐ My patient asked about it
- ☐ *Neurology*® journal
- ☐ *Neurology Now*®
- ☐ *Neurology Today*®
- ☐ News media source (please identify): ______________________________
- ☐ Pinterest
- ☐ Twitter
- ☐ Website (please identify): ______________________________
- ☐ Your institution (please identify): ______________________________
- ☐ YouTube
- ☐ Other (please describe):______________________________

13. Have you read the AAN guideline or physician/patient summary on CAM use in MS?
- ☐ Yes
- ☐ No → **Please continue with Question 14 on page 7.**

13a. What sources did you use to read or access the guideline?
Please check all that apply.
- ☐ AAN website
- ☐ E-mail sent to me
- ☐ Google+
- ☐ Facebook
- ☐ LinkedIn
- ☐ *Neurology* journal
- ☐ *Neurology Now*
- ☐ *Neurology Today*
- ☐ Pinterest
- ☐ Twitter
- ☐ Website (please identify): ______________
- ☐ YouTube
- ☐ Other (please describe): ______________________________

14. Do you ***plan to initiate discussions*** *about* any of these CAM therapies with your patients with MS? ***Please check all that apply*** *or select "No" if you do not plan on discussing any CAM therapies.*

◻ **No, I do not plan to initiate any discussions about any CAM therapies with my patients with MS.**

- ☐ Acupuncture
- ☐ Amalgam replacement
- ☐ Bee venom (sting)
- ☐ Biofeedback
- ☐ Cannabis extract (e.g., Cesamet, Marinol)
- ☐ Carnitine
- ☐ Chelation
- ☐ Chiropractic care
- ☐ Creatine
- ☐ Ginkgo biloba
- ☐ Glucosamine
- ☐ Hippotherapy (therapeutic horseback riding)
- ☐ Hyperbaric oxygen
- ☐ Inosine
- ☐ Linoleic acid
- ☐ Lofepramine
- ☐ Low-dose naltrexone
- ☐ Magnetic therapy
- ☐ Massage therapy
- ☐ Mindfulness
- ☐ Music therapy
- ☐ Naturopathic medicine
- ☐ Neural therapy
- ☐ Omega-3 fatty acid supplementation (e.g., fish oil)
- ☐ Padma 28
- ☐ Phenylalanine
- ☐ Progressive muscle relaxation
- ☐ Reflexology
- ☐ Smoking marijuana
- ☐ Tai chi
- ☐ Threonine
- ☐ Transdermal histamine (e.g., Prokarin patch)
- ☐ Yoga
- ☐ Other (please describe): ___________________________________ ___________________________________

15. Since *March 2014*, have you advised patients ***to start using or stop using*** any of these CAM therapies for MS? *Please check all that apply for both questions.*

	I have advised patients to ***start using*** this therapy.	I have advised patients to ***stop using*** this therapy.
Acupuncture	☐	☐
Amalgam replacement	☐	☐
Bee venom (sting)	☐	☐
Biofeedback	☐	☐
Cannabis extract (e.g., Cesamet, Marinol)	☐	☐
Carnitine	☐	☐
Chelation	☐	☐
Chiropractic care	☐	☐
Creatine	☐	☐
Ginkgo biloba	☐	☐
Glucosamine	☐	☐
Hippotherapy (therapeutic horseback riding)	☐	☐
Hyperbaric oxygen	☐	☐
Inosine	☐	☐
Linoleic acid	☐	☐
Lofepramine	☐	☐
Low-dose naltrexone	☐	☐
Magnetic therapy	☐	☐
Massage therapy	☐	☐
Mindfulness	☐	☐
Music therapy	☐	☐
Naturopathic medicine	☐	☐
Neural therapy	☐	☐
Omega-3 fatty acid supplementation (e.g., fish oil)	☐	☐
Padma 28	☐	☐
Phenylalanine	☐	☐
Progressive muscle relaxation	☐	☐
Reflexology	☐	☐
Smoking marijuana	☐	☐
Tai chi	☐	☐
Threonine	☐	☐
Transdermal histamine (e.g., Prokarin patch)	☐	☐
Yoga	☐	☐
Other (please describe): ___________________________ ___________________________	☐	☐

16. Do you discuss AAN guideline recommendations with your patients with MS?
   - ☐ Yes → 16a
   - ☐ No → **Please continue with Question 17.**

> 16a. When you speak with patients about guideline-related information, do you use or distribute guideline summaries or tools for patients and their families?
> - ☐ Yes
> - ☐ No

17. Because of new information gained from AAN guidelines on the use of CAM in MS, have you changed your practice regarding discussing CAM therapies with your patients with MS?
   - ☐ Yes, I have changed my practice → **Please go to Question 17a.**
   - ☐ No, I do not do anything different → **Please go to Question 18 on Page 10.**

> 17a. Please describe how you have changed your practice.
> ____
> ____
> ____

The final set of questions is about you and your practice.

18. How old are you?
    ________ years old

19. What is your gender?
    ☐ Female
    ☐ Male
    ☐ Prefer not to answer

20. Do you consider yourself Hispanic or Latino/a?
    ☐ Yes, Hispanic or Latino/a
    ☐ No

21. What is your race? ***Please check all that apply.***
    ☐ American Indian or Native American
    ☐ Asian
    ☐ Black or African American
    ☐ Hawaiian or other Pacific Islander
    ☐ White
    ☐ Other (please specify):
    ____

22. What is your practice focus?
    ☐ Solo practice
    ☐ Neurology group
    ☐ Multispecialty group
    ☐ University-based group
    ☐ Staff-model HMO
    ☐ Government hospital or clinic
    ☐ Other public/private hospital clinic (please specify):
    ____
    ☐ No clinical practice

23. How many years have you been in practice?
    ☐ 0–5 years
    ☐ 6–10 years
    ☐ 11–15 years
    ☐ 16–20 years
    ☐ 21–25 years
    ☐ 26–30 years
    ☐ 31+ years

24. How many patients are you presently treating for their symptoms of MS?
    ☐ 0–10 patients with MS
    ☐ 11–20 patients with MS
    ☐ 21–30 patients with MS
    ☐ 31–40 patients with MS
    ☐ 41–50 patients with MS
    ☐ 51+ patients with MS

Thank you for your feedback!
Please return your survey in the self-addressed return envelope.
