# Supplementary material for: The Impact of Social Media on Dissemination and Implementation of Clinical Practice Guidelines: A Longitudinal Observational Study
Source: J Med Internet Res. 2015 Aug 13;17(8):e193. doi: 10.2196/jmir.4414 (PMC4736287; doi:10.2196/jmir.4414)
Supplement: Multimedia Appendix 4 [file jmir_v17i8e193_app4.pdf]

MULTIMEDIA APPENDIX 4: RESULTS OF DISSEMINATION EFFORTS:

The number of people viewing the CPG was captured by clicks on links, time spent on webpages, forwards of e-mails, news clips, publicity value, and impressions. An impression is defined as the number of subscribers to a particular media outlet or followers that an individual or organization has on its social media channels. Publicity value is defined as the cost of advertising in a media outlet multiplied by a factor of 3.

Traditional Dissemination: In the three month period of traditional dissemination, the guideline was accessed 17,499 times at neurology.org. One thousand seven hundred eighty-five individuals (2,021 total access) accessed aan.com/guidelines/home, spending an average of 1:49 minutes on the website. The most frequent ways that individuals accessed this site were via a search on Google's search engine (1,221), a direct search (331), Microsoft's Bing search engine (92), and through AAN's e-mail distribution client, Informz (64). Four thousand sixty-seven individuals (5,334 total accesses) accessed aan.com/guidelines/home/multiple sclerosis, spending an average of 2:53 minutes on the website. The most frequent ways that individuals accessed this site were similar to the above: Google's search engine (3,408), through Informz (561), a direct search (439), and Bing search engine (143).

Of the 26,517 member e-mails, 7,809 (29%) were opened. Of those, 919 (12%) individuals accessed the link to the CPG within the e-mail and 485 (6%) forwarded the e-mail.

The press release page of AAN.com received 317 unique page views (358 in all) with an average of 2:46 minutes spent on the page. The press release and public relations efforts

generated 2,849 news clips including US News & World Report, NBC News, CBS News, CNN, and the LA Times. These news clips generated 2,621,885,408 impressions with a calculated publicity value of $2,978,147.63.

Novel Dissemination: The *Neurology*® podcast discussing the CPG conclusions and recommendations was downloaded 38,155 times. The patient and physician informational videos on YouTube have been viewed 72,262 and 650 times respectively. Over the 90 day period Facebook generated 1,200,000 impressions through 40,337 clicks, Twitter generated 457,222 impressions through 9,410 clicks, YouTube generated 559,730 impressions with 3,746 clicks, and LinkedIn generated 243,018 impressions with 601 clicks. During the hour of the Tweetchat, nearly 5,000,000 impressions were generated. We linked the digital advertisements to the summary PDF of the guideline. While we can't track downloads, we assume that the viewer may have read the summary by virtue of clicking the advertisement, although we cannot determine the extent to which the guideline contents were read.
